# Baseline GABA+ levels in areas associated with sensorimotor control predict initial and long‐term motor learning progress

**DOI:** 10.1002/hbm.26537

**Published:** 2023-12-22

**Authors:** Hong Li, Sima Chalavi, Amirhossein Rasooli, Geraldine Rodríguez‐Nieto, Caroline Seer, Mark Mikkelsen, Richard A. E. Edden, Stefan Sunaert, Ron Peeters, Dante Mantini, Stephan P. Swinnen

**Affiliations:** ^1^ Movement Control and Neuroplasticity Research Group Group Biomedical Sciences, KU Leuven Leuven Belgium; ^2^ KU Leuven Brain Institute (LBI), KU Leuven Leuven Belgium; ^3^ Department of Radiology Weill Cornell Medicine New York New York USA; ^4^ Russell H. Morgan Department of Radiology and Radiological Science Johns Hopkins University School of Medicine Baltimore Maryland USA; ^5^ F. M. Kirby Research Center for Functional Brain Imaging Kennedy Krieger Institute Baltimore Maryland USA; ^6^ Department of Imaging and Pathology KU Leuven and University Hospital Leuven (UZ Leuven) Leuven Belgium

**Keywords:** augmented visual feedback, GABA, Glx, internally‐guided and externally‐guided movement, motor learning, MRS, proprioception

## Abstract

Synaptic plasticity relies on the balance between excitation and inhibition in the brain. As the primary inhibitory and excitatory neurotransmitters, gamma‐aminobutyric acid (GABA) and glutamate (Glu), play critical roles in synaptic plasticity and learning. However, the role of these neurometabolites in motor learning is still unclear. Furthermore, it remains to be investigated which neurometabolite levels from the regions composing the sensorimotor network predict future learning outcome. Here, we studied the role of baseline neurometabolite levels in four task‐related brain areas during different stages of motor skill learning under two different feedback (FB) conditions. Fifty‐one healthy participants were trained on a bimanual motor task over 5 days while receiving either concurrent augmented visual FB (CA‐VFB group, *N* = 25) or terminal intrinsic visual FB (TA‐VFB group, *N* = 26) of their performance. Additionally, MRS‐measured baseline GABA+ (GABA + macromolecules) and Glx (Glu + glutamine) levels were measured in the primary motor cortex (M1), primary somatosensory cortex (S1), dorsolateral prefrontal cortex (DLPFC), and medial temporal cortex (MT/V5). Behaviorally, our results revealed that the CA‐VFB group outperformed the TA‐VFB group during task performance in the presence of augmented VFB, while the TA‐VFB group outperformed the CA‐VFB group in the absence of augmented FB. Moreover, baseline M1 GABA+ levels positively predicted and DLPFC GABA+ levels negatively predicted both initial and long‐term motor learning progress in the TA‐VFB group. In contrast, baseline S1 GABA+ levels positively predicted initial and long‐term motor learning progress in the CA‐VFB group. Glx levels did not predict learning progress. Together, these findings suggest that baseline GABA+ levels predict motor learning capability, yet depending on the FB training conditions afforded to the participants.

## Graphical abstract

Neurometabolites, such as gamma‐aminobutyric acid (GABA) and glutamate (Glu), play a critical role in synaptic plasticity and learning. In this study, we studied the role of baseline neurometabolite levels in four task‐related brain areas during different stages of motor skill learning under two different feedback (FB) conditions. Our findings suggest that baseline GABA+ constitutes a potential biomarker for motor learning capacity in young adults and this depends on the FB training conditions afforded to the participants.

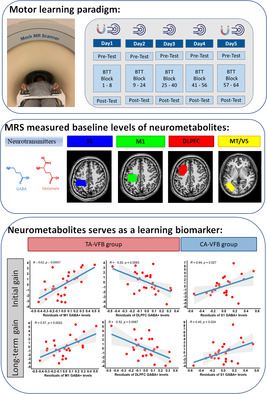

## 1 INTRODUCTION

Skill learning is a critical part of our existence and is even instrumental to survival. It refers to an internal process, which benefits from experience or practice and leads to relatively permanent changes in the capability of skilled movement production (Schmidt et al., 2019). To effectively study motor learning, it is important to distinguish between the terms “motor performance,” which is an observable behavior, and “motor learning,” which is not immediately observable but can indirectly be inferred from the observation of performance under certain circumstances (Magill & Anderson, 2017). It is important to note that the observed performance during practice may overestimate or underestimate the actual amount of learning achieved (Magill & Anderson, 2017). For example, the transient changes in behavior during training with various types of augmented feedback (such as verbal or visual information about performance) may alter or vanish in the absence of such feedback (FB) and overestimate learning. Alternatively, mental and physical fatigue may suppress performance levels temporarily during practice and can thus underestimate the amount of learning. Such temporary changes in behavior do not reflect motor learning, because they are not sufficiently permanent (Salmoni et al., 1984). A possible solution is to set up test conditions in which performance is assessed while these temporary effects have vanished.

Although there is no gold standard to characterize the different stages of motor learning, a division between initial and later learning is often made (Coynel et al., 2010; Gentile, 1972, 1987, 2000). During initial learning, the performer explores the most effective strategies and builds neuromuscular patterns. The later stage is characterized by subtle adjustments while the movements become more efficient and consistent, leading to automaticity. The learning rate is typically high during this initial stage and reduces during the later stage when performance reaches a plateau. In spite of this, learning unfolds as a continuous transition from early to later stages.

Various types of FB have been studied to facilitate or optimize motor skill acquisition (Hantzsch et al., 2022; St Germain et al., 2022; Swinnen, 1996). It is commonly agreed that different features of FB, such as its frequency and timing, can be used to guide performance and learning (Salmoni et al., 1984). Specifically, performance is typically better in the presence of concurrent FB (delivered during ongoing movement) as it provides direct online guidance to adjust performance and minimize errors. Conversely, instant performance is relatively poorer when it is provided after the completion of the task (i.e., terminal FB), as no direct guidance is provided during the task execution itself. But this reflects performance and not necessarily learning effects. Moreover, when the augmented FB is withdrawn, performance may deteriorate. This has been coined as the “guidance hypothesis of information FB,” suggesting that FB may have a dual function: FB presence can boost performance instantaneously but it can also hamper learning, as assessed under FB withdrawal (FBW) conditions (Salmoni et al., 1984; Schmidt, 1991).

While enormous investments have been made during the past decades to understand and optimize motor learning through FB and variations of the training context, recent research has focused on the role of neurometabolites in relation to (motor) learning. This is inspired by evidence showing the practice‐induced formation of new neural connections and/or the strengthening of existing ones through a process known as synaptic plasticity (Carcea & Froemke, 2013). Synaptic plasticity highly relies on the balance between excitation and inhibition in the brain (Carcea & Froemke, 2013; Dorrn et al., 2010). As the primary inhibitory and excitatory neurotransmitters, gamma‐aminobutyric acid (GABA) and glutamate (Glu), play critical roles in synaptic plasticity and learning. The advent of magnetic resonance spectroscopy (MRS) has allowed accurate and in vivo quantification of the concentrations of brain neurometabolites, such as GABA and Glu (Puts & Edden, 2012). Because the MRS‐measured GABA levels contain a significant contribution from macromolecules (GABA+ macromolecules), we refer to the resulting measure as GABA+ levels. Additionally, because it is often not possible to distinguish Glu from glutamine in 3 T MRI systems, we refer to the resulting measure targeted at Glu as Glx (Glu + Glutamine).

So far, numerous studies have investigated whether MRS‐measured baseline (resting state) levels of GABA+ and Glx are linked to various behavioral “performance” metrics pertaining to cognitive, perceptual, and motor tasks (Li et al., 2022; Pasanta et al., 2023). Depending on the type of task, neurochemical levels can be positively (Mikkelsen et al., 2018; Puts et al., 2015) or negatively (Marsman et al., 2017; Takei et al., 2016) related to behavioral performance. However, studies addressing the relationship between baseline GABA+ levels and “learning” are still very scarce.

Studies on the associations between MRS‐assessed baseline neurometabolite levels and motor learning gain have reported mixed findings. One study that used a serial finger tapping task revealed that lower baseline levels of GABA+ in the primary motor cortex (M1) were associated with greater subsequent motor learning (Kolasinski et al., 2019). However, other motor learning studies making use of a finger sequencing task (Stagg et al., 2011) and a bimanual tracking task (BTT; Chalavi et al., 2018) demonstrated lower baseline M1 GABA+ levels to be related to better initial performance, even though no significant correlations were observed between baseline levels of M1 GABA+ or Glx and learning measures.

Our goal was to determine whether baseline levels of neurometabolites are associated with learning gain during different stages of motor learning. This prompted questions about which brain regions to select. Motor skill relies on a distributed network of cortical and subcortical regions and practice leads to functional changes in these areas, including increases, decreases, or no consistent changes in brain activity (Debaere et al., 2004; Doyon et al., 1998; Puttemans et al., 2005; Rémy et al., 2008). Moreover, the learning‐related brain changes also depend on the type of FB that is made available during task performance (Beets et al., 2015; Debaere et al., 2003; Ronsse et al., 2011). This complicates the choice of brain regions for neurochemical investigation of motor learning.

In relation to bimanual skill learning, some brain regions are more activated during the initial stage of learning while others become more active during the later stages (Debaere et al., 2004; Rémy et al., 2008). For example, the primary motor (M1) and secondary motor areas are involved in movement planning and production and remain active throughout learning. In interaction with M1, the somatosensory (S1) cortex processes task‐specific information about self‐movement and body position (also known as proprioception). Other regions play a more temporary role, such as the dorsolateral prefrontal cortex (DLPFC) which is typically activated during the initial learning stage while showing a reduction of activity at later stages (Debaere et al., 2004; Rémy et al., 2008). Conversely, the striatum and cerebellum contribute to the automatization process and show enhanced subregional activity during the later learning stages. Furthermore, when augmented visual FB (VFB) is provided during task performance and/or learning, occipital areas kick in, such as the primary visual cortex (V1) and associated regions, but also parietal and medial temporal cortex, including MT/V5, a region specialized in motion processing (Debaere et al., 2004; Ronsse et al., 2011). Thus far, MRS studies on motor function have primarily focused on the sensorimotor cortex while other task‐related areas have yet to be investigated.

Here, we used a bimanual task consisting of several subtasks. Our working hypothesis was that neurometabolite levels may predict skill learning capability. Our principal aims were to (1) investigate the relationship between baseline (resting state) levels of neurometabolites from a selection of task‐related brain areas and motor learning gain during different stages of motor learning, and (2) assess whether the learning gain obtained under different types of augmented VFB is related to baseline levels of neurometabolites in the FB‐processing brain areas. Specifically, one group was provided with concurrent augmented VFB (CA‐VFB group), implying that participants could see the real‐time visual FB of their movement together with the template of the correct movement on a PC screen during task execution (externally‐ or visually‐generated movement). The second group was provided with Terminal Augmented VFB (TA‐VFB group). Hence, participants could only see their movement trajectory on a PC screen on top of the ideal typical trajectory after trial completion, that is, they relied on the emerging proprioceptive information from actual task performance (internally‐ or proprioceptively‐generated movement). Irrespective of the training group, all participants were subjected to the same tests before the start of the training (pre‐test) and after the end of the training (post‐test) on each day. In order to eliminate the temporary effects of augmented VFB, these tests were always performed in the absence of any augmented VFB (either during or after task performance) in both groups to assess true learning gains across days under comparable conditions for both groups.

First, from a *behavioral perspective*, we hypothesized that the CA‐VFB group would improve rapidly and show better task performance during training than the TA‐VFB group because the former could continuously adjust performance based on the real‐time FB during task execution. However, from a learning perspective, we anticipated that the CA‐VFB group would perform worse during the no‐FB test conditions than the TA‐VFB group because participants of the former group had become dependent on the visual FB such that their performance became vulnerable when weaning from this FB.

Second, from a neurometabolite‐behavioral perspective, we hypothesized that both groups would benefit from higher GABA+ levels in the M1 to lay down the distinct subtask representations in motor memory across learning. Similarly, we predicted that higher baseline DLPFC GABA+ levels would support the built‐up of these representations, particularly during initial learning. In view of the differential FB manipulation according to the group, we hypothesized that higher GABA+ levels in the MT/V5 would support performance in the CA‐VFB group because of the dominant role of concurrent visual FB during training (vision‐based learning). Alternatively, we hypothesized that the TA‐VFB group would show higher learning gains with higher GABA+ levels in the S1 because the absence of concurrent visual FB would leave them with somatosensory input from S1 as the only source of reliable information for generation of the movement representations (proprioception‐based learning). In general, we anticipated that the role of these neurometabolite levels would be more critical during the early than late stage of learning because of the critical role of sensory information during early learning. Finally, we also measured baseline Glx levels in the same brain regions to determine their role in motor learning in comparison with GABA.

## 2 MATERIALS AND METHODS

### 2.1 Participants

Here, 57 young adults (28 females, aged 18–34 years, mean ± SD = 25.53 ± 4.04) initially participated in this study. Participants had normal or corrected‐to‐normal vision and reported no history of neurological disease or psychiatric disorders. Participants were randomly assigned to one of the two groups: CA‐VFB group or TA‐VFB group. The demographic information of both groups, including age, gender, and handedness, is presented in Table 1. Groups did not significantly differ with respect to age (independent t‐test: *t* = −0.15, *df* = 55, *p* = .88), gender (*χ* ^*2*^ = 0.15, *df* = 1, *p* = .90), and handedness (Wilcoxon rank‐sum test: *w* = 378, *p* = .64) (Oldfield, 1971). Informed consent was obtained from all participants before they entered the research. The study protocol was in accordance with the declaration of Helsinki (1964) and was approved by the Ethics Committee Research of UZ/KU Leuven (study number S58333 and its amendment).

**TABLE 1:** Demographic information and experiment time across 5 days.

	TA‐VFB group	CA‐VFB group	Statistic	*p*‐Value
*Demographics*				
Age (mean ± SD)	25.3 2 ± 4.3	25.26 ± 3.8	*t* = 0.04	.96
Gender (M/F)	13/12	13/13	*χ* ^2^ = 0.02	.89
Handedness	97%	96%	*w* = 290	.49
*Experiment time*				
Day 1 (morning/afternoon)	12/14	12/13	*χ* ^2^ = 0.02	.89
Day 2 (morning/afternoon)	9/17	10/15	*χ* ^2^ = 0.16	.69
Day 3 (morning/afternoon)	9/17	13/12	*χ* ^2^ = 1.57	.21
Day 4 (morning/afternoon)	8/18	9/16	*χ* ^2^ = 0.16	.69
Day 5 (morning/afternoon)	10/16	12/13	*χ* ^2^ = 0.47	.49

*Note*: The handedness value is measured by the Edinburgh Handedness Inventory (Oldfield, 1971).

Abbreviations: F, female; M, male.

### 2.2 Overview of experiment sessions

**FIGURE 1:**
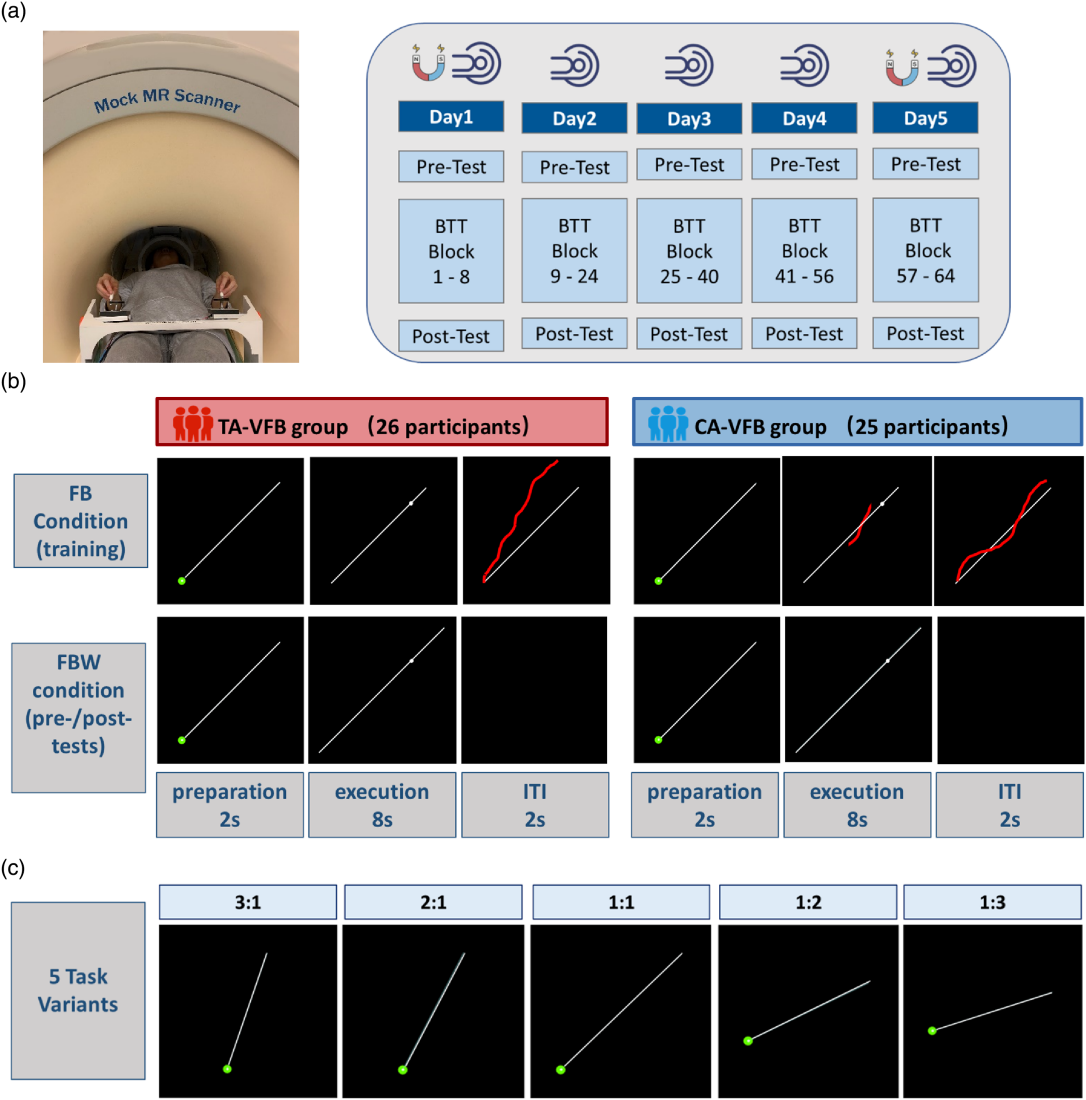
(a) Bimanual setup and behavioral training protocol. On the first (Day 1) and last (Day 5) days of training, the trials were performed in the actual MRI scanner while during the remaining training days, the trials were performed inside a mock scanner. (b) FB (training) and FBW (pre‐tests and post‐tests) conditions in both TA‐VFB group and CA‐VFB group. (c) The five task variants in each task block. CA‐VFB group, concurrent augmented visual feedback group; FB condition, feedback condition; FBW condition, feedback withdrawal condition; ITI, inter‐trial interval; TA‐VFB group, terminal augmented visual feedback group.

This study consisted of one screening session and five behavioral training sessions which were spread across a time window of 7.9 ± 0.5 days (mean ± SD) (Figure 1a). During the screening session (Day 0), participants' handedness was assessed and contra‐indications to MRI were determined. Then, they were familiarized with the behavioral task as well as the MR scanner environment. Day 0 was followed by 5 days of training on a bimanual task (i.e., Day 1 to Day 5). Each training day started with a pre‐test, followed by a training part, and ended with a post‐test. The pre‐test and post‐test were performed in the absence of any type of augmented VFB (i.e., the total FBW condition) while the training part was performed in the presence of visual FB (i.e., the FB condition). The FBW condition was determined as the critical test of learning and was the same for both groups. As such, the latter test conditions constituted a possible advantage for participants of the TA‐VFB group who were more familiar with such a context during training as compared to the CA‐VFB group who were deprived of the real‐time augmented VFB during the tests. Figure 1b illustrates the stimuli that were presented to the participants during FB (training) and FBW (pre−/post‐test) conditions. On the first (Day 1) and last (Day 5) days of training, the trials were performed inside the actual MRI scanner while during the remaining training days, the trials were performed inside a mock scanner, in order to mimic the MR scanner environment. We categorized the timing of the experiment into two segments: morning (experiment initiated before 12:00 PM) or afternoon (experiment initiated at or after 12:00 p.m.). There was no significant difference in experimental time across the five training sessions between the groups (Table 1).

### 2.3 Bimanual tracking task

#### 2.3.1 BTT description

Participants laid in a supine position inside the actual or mock MR scanner. The task device, which consisted of two dials (diameter of 5 cm) for movement recording, was positioned over the participants' laps and fixated in the lateral ramps of the MRI table (Sisti et al., 2011). Visual stimuli, which consisted of a white dot moving along a blue straight target template, were projected onto either a double mirror placed in front of the participants' eyes (inside the actual MR scanner) or a screen in front of the participants' eyes (inside the mock MR scanner). Participants were instructed to closely track the white dot on the screen, by rotating the two dials simultaneously. The left dial controlled the displacement along the y‐axis (clockwise: upward, counter‐clockwise: downward) and the right dial along the x‐axis (clockwise: right, counter‐clockwise: left). Each trial consisted of a preparation phase (2 s), an execution phase (8 s) and an inter‐trial interval (ITI) of 2 s (Figure 1b). During the preparation phase, the target template was visualized but no movement was required, and the participants were instructed to plan their movement. The start of the execution phase was marked by the appearance of a white target dot that started moving along the target template. During the execution phase, participants were instructed to track the white target dot as accurately as possible both spatially and temporally.

#### 2.3.2 BTT schedule and FB conditions

On Day 0, the target template consisted of a straight line with an angle of 45°, requiring a rotation of both dials at equal speed. Participants completed a familiarization block of eight trials including two in each movement orientation (upward‐right, upward‐left, downward‐right and downward‐left). From Day 1 to Day 5, participants in both groups practiced five movements with different frequency ratios (Figure 1c), that is, 3:1, 2:1, 1:1, 1:2, and 1:3 (left hand:right hand), only in one orientation (upward‐right, both hands clockwise) during the training and pre‐ and post‐tests. The frequency ratio of 1:1 refers to the same rotation speed of both hands (less challenging) while the remaining frequency ratios refer to different cooperative speeds of the two hands (more challenging). Accordingly, learning of this skill required participants to generate five distinct subtask representations.

During the training part, which was performed in the presence of augmented *FB* (*FB condition*), participants received a specific type of visual FB according to their group assignment. Specifically, in the CA‐VFB group, augmented VFB was provided by displaying the ongoing trajectory of participants' movement in real time on a PC screen (red dot) such that they could adjust their trajectory based on the visual FB. However, in the TA‐VFB group, no concurrent visual FB was provided during actual task performance but participants observed their full movement trajectory after completion of each trial (i.e., during ITI) to support task acquisition. During the training part, participants completed 120 trials on Day 1 and Day 5, and 240 trials on Day 2 to Day 4, with equal numbers of trials in each ratio. The number of trials was limited during the actual scanning days (Day 1 and Day 5) because of the time constraint. On each training day, a pre‐test and a post‐test (10 trials, including 2 trials/ratio) were administered before and after training. During pre‐test and post‐tests, no augmented FB was provided during or after the trial to either of the groups (*FBW condition*). This served as the ultimate test condition for the assessment of learning because temporary effects of FB provision were eliminated and both groups were tested under exactly the same conditions.

#### 2.3.3 BTT analysis

Behavioral data were recorded and analyzed with LabVIEW. The x‐ and y‐coordinates of the target dot and the participants' cursor positions were sampled at 100 Hz. Offline data analysis was carried out using MATLAB 2021. The performance accuracy level of each trial was assessed by calculating the tracking deviation (TD), based on the average track deviation. That is, for each trial, the track deviation was measured as the distance between the target dot and participants' cursor position at each point in time and subsequently averaged. The behavioral data from six participants were excluded since either they were not able to complete the whole behavioral training program because of personal reasons (*n* = 4) or the behavioral performance was identified as an outlier (*n* = 2). Accordingly, for the behavioral analysis, we proceeded with the complete datasets obtained from 51 participants (CA‐VFB group [*n* = 25]; TA‐VFB group [*n* = 26]). Among these 51 participants, there was no difference between the CA‐VFB and TA‐VFB groups with respect to age (independent *t* test: *t* = 0.04, *p* = .96), gender (*χ* ^2^ = 0.02, *p* = .89) and handedness (Wilcoxon rank‐sum test: *w* = 290, *p* = .49).

### 2.4 Magnetic resonance spectroscopy

#### 2.4.1 MRI data acquisition

**FIGURE 2:**
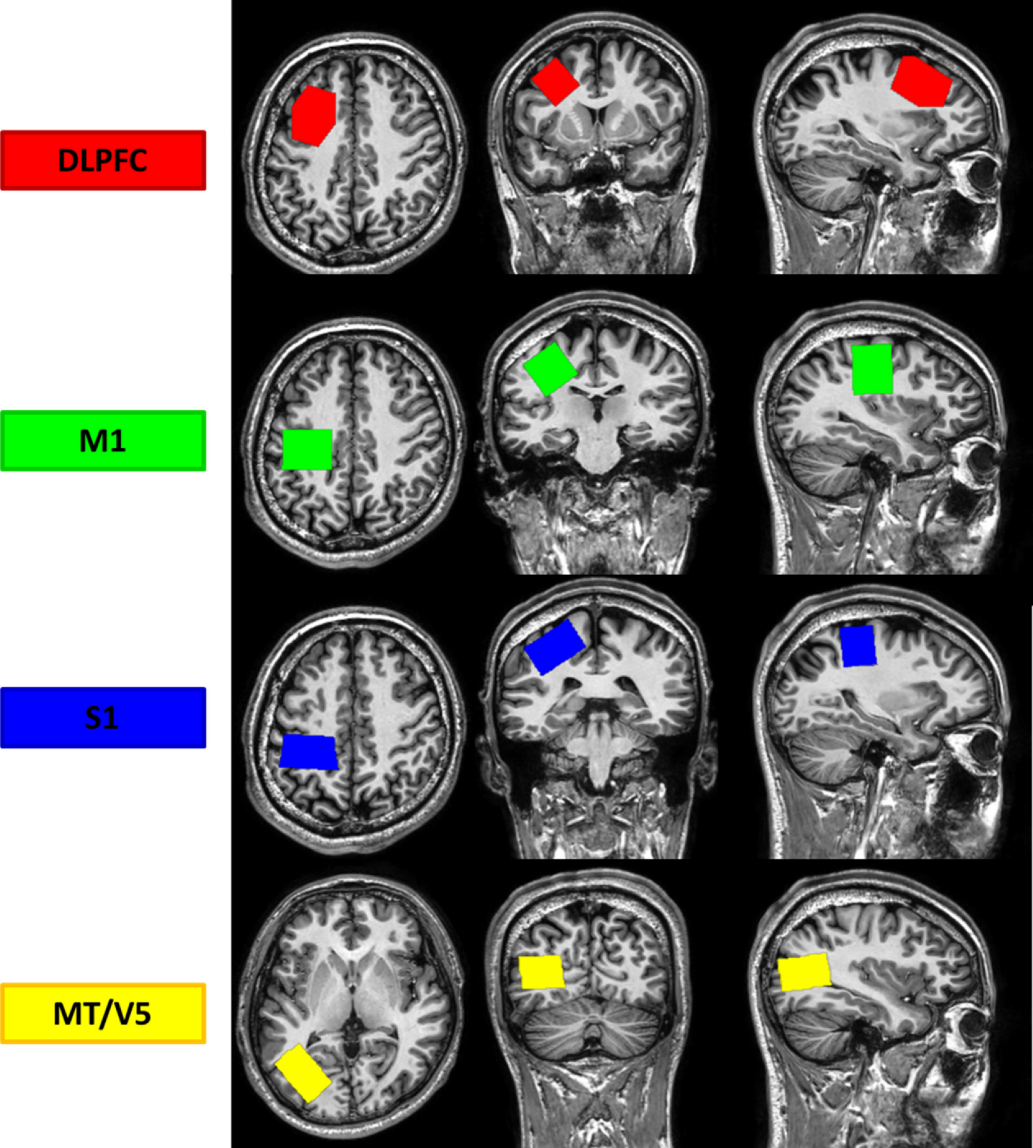
MRS voxel positioning. An example of placement of each VOI on the anatomical image of a participant. Color coding: DLPFC VOI in red, M1 VOI in green, S1 VOI in blue, and MT/V5 VOI in yellow.

MRI data were acquired using a 3 Tesla Philips Achieva scanner with a 32‐channel receiver head coil (University Hospital Leuven, Gasthuisberg). At the beginning of the MR session, a high‐resolution *T*_1_‐weighted anatomical image was acquired using a chemical shift three‐dimensional turbo field echo (3DTFE) (TE = 4.6 ms, TR = 9.7 ms, 1 mm^3^ voxel size, field of view = 256 × 242 × 182 mm^3^, 182 sagittal slices, scan duration = ~6 min). MRS data were acquired using the MEGA‐PRESS sequence (Edden & Barker, 2007; Mescher et al., 1998) (TE = 68 ms, TR = 2 s, 2 kHz spectral width). In light of the pivotal role of the left hemisphere in controlling bilateral movements (Merrick et al., 2022), all of the MRS voxels were placed in the left hemisphere for all participants. Considering the shape and dimensions of each region of interest and based on previous studies, voxel dimensions were set to 30 × 30 × 30 mm^3^ for the M1 volumes of interest (VOI) (Maes et al., 2018), whereas the dimensions of the DLPFC and MT/V5 voxels were set to 40 × 25 × 25 mm^3^ (Greenhouse et al., 2016) and the dimensions of the S1 voxels were set to 25 × 40 × 25 mm^3^. For the DLPFC, S1, and MT/V5 voxels, 160 averages were acquired (scan time = 11 min 12 s). However, since the number of averages can be reduced without affecting the data quality for the M1 voxel (Mikkelsen et al., 2018), 112 averages were acquired for the left M1 VOI voxel (scan time = 8 min). ON and OFF spectra were acquired in an interleaved fashion, corresponding to an editing pulse at 1.9 or 7.46 ppm, respectively. Prior to each MRS acquisition, an automatic shimming procedure was performed. For each MRS VOI, 16 unsuppressed water averages were acquired within the same VOI using identical acquisition parameters. MRS VOIs were identified on a subject‐to‐subject basis using anatomical landmarks (Figure 2). The M1 VOI was placed over the hand knob of the motor cortex and in line with the cortical surface in the sagittal plane (Yousry et al., 1997). Similarly, the S1 VOI was first placed over the hand knob of the motor cortex and then moved in a posterior direction until it covered the postcentral gyrus. This voxel was also aligned with the cortical surface in the coronal plane. For the DLPFC voxel, first the center of the voxel was positioned in the axial slice above the superior margin of the lateral ventricles. In this slice, the DLPFC voxel was placed at one third of the anterior‐to‐posterior distance of the brain, centered in between the lateral and medial wall of each hemisphere (Maes et al., 2021; O'Gorman et al., 2011). Afterward, it was also visually inspected whether the DLPFC voxel properly covered the middle frontal gyrus. For placement of the MT/V5 VOI, the anatomical slices were first screened from lateral to medial on the sagittal view, and then the center of the voxel was placed at the end of the medial temporal gyrus (the conjunction between temporal and occipital cortex) while ensuring that the lateral sides are in line with the cortical surface in the sagittal and axial planes. Figure S1 shows the heatmap of the locations of MRS VOIs and the MRS spectra obtained from these VOIs in two groups. Of note, MRS requires the use of relatively large voxels to ensure an acceptable signal‐to‐noise ratio (SNR) (Mullins et al., 2014). Therefore, since the M1 and S1 VOIs are localized in the vicinity of each other, some overlap was expected. Importantly, the center of each VOI was placed independently, ensuring that the overlap between VOIs was reduced. Following the completion of data collection, we quantitatively inspected the extent of the S1‐M1 overlap rate in each participant by calculating the volume of the S1‐M1 overlap and dividing it once by the volume of the M1 voxel and once by the volume of the S1 voxel. Results of this analysis revealed that the mean overlap rate with the M1 VOI was 46% in the TA‐VFB group and 44.5% in CA‐VFG group, and the mean overlap rate with the S1 VOI was 49.9% in the TA‐VFB group and 48.2% in the CA‐VFG group. Importantly, the overlap rates were not statistically different between groups (Table 2 and Figure S2).

**TABLE 2:** Overlap rate between the S1 and M1 voxels.

	Mean	SD	*t*‐test	*df*	*p*‐Value
*Overlap* (*% M1*)					
TA‐VFB group	46.0%	7.37	0.700	49	.487
CA‐VFB group	44.5%	7.64			
*Overlap* (*% S1*)					
TA‐VFB group	49.9%	7.95	0.752	49	.456
CA‐VFB group	48.2%	8.27			

Abbreviations: CA‐VFB group, concurrent augmented visual feedback group; M1, primary motor cortex; S1, primary somatosensory cortex; TA‐VFB group, terminal augmented visual feedback group.

During Day 1 and Day 5, MRS data from S1 and MT/V5 were collected at three timepoints: before (resting state), during (task‐related), and after the training (resting state). Additionally, on Day 1, before the start of the training, additional MRS data were acquired from the M1 and DLPFC during the resting state. Please note that the MRS data obtained during and after the behavioral task on Day 1 as well as the MRS data obtained on Day 5 will not be discussed in the current manuscript.

#### 2.4.2 MRS data processing

MRS data were analyzed using the Gannet toolkit (version 3.2.1) (Edden et al., 2014). In the first step, data were frequency‐and‐phase‐corrected by applying spectral registration (Mikkelsen et al., 2020). The ON spectra were subtracted from the OFF spectra, and the resulting difference spectrum was fitted between 4.2 and 2.8 ppm using a three‐Gaussian function. The water signal was fitted using a Lorentz‐Gaussian model and it was used as the reference metabolite. Subsequently, MRS voxels were co‐registered to the individual anatomical image, and statistical parametric mapping (version 12) was used to segment brain tissues inside the VOIs into different tissue fractions (gray matter, white matter, and cerebrospinal fluid). These tissue fractions were used to correct the obtained GABA+ levels for partial volume effects, with the assumption that GABA is absent in cerebrospinal fluid and has a concentration that is twice as high in gray as compared to white matter (Harris et al., 2015, equation 5). Finally, as there was no reason to assume any differences in the brain parameters between the two groups of young adults, GABA+ levels were normalized to the average voxel composition of both groups combined (Harris et al., 2015, equation 6). Data quality was assessed by visual inspection of the spectra for lipid contamination, poor water suppression and by examining the fit error and SNR (Mullins et al., 2014). Ultimately, GABA+ levels were obtained from all four brain VOIs of 57 participants. However, for the Glx levels, measurement from the left M1 voxel from one participant was excluded due to insufficient data quality. An overview of the MRS data quality measures is provided in the supplementary Table S1.

### 2.5 Statistical analysis

Statistical analyses were carried out using R (4.1.2). First, we assessed whether the assumptions of parametric statistical tests, such as normality and homogeneity, were met. If so, parametric statistical tests were used. If not, nonparametric alternatives were used.

#### 2.5.1 BTT data analysis

##### BTT performance

First, we used a t‐test to investigate whether initial performance (Day 1 pre‐test) was different between groups. Then we assessed whether behavioral performance improved with training and whether the two groups improved differently. To do so, the BTT performance data during the *FB‐supported condition* (training) were analyzed using a nonparametric alternative of two‐way mixed 2 (Groups) × 5 (Days) ANOVA model. Furthermore, to test learning gains, the data during the *FBW condition* (pre‐ and post‐tests) were analyzed using a nonparametric alternative of three‐way mixed ANOVA as 2 (Groups) × 5 (Days) × 2 (Pre − Post) model using “nparLD” in R (http://www.R205 project.org) (Noguchi et al., 2012, 2020). In addition, to investigate whether final performance in the short‐term or long‐term (Day 1 post‐test or Day 5 post‐test) differed between groups, additional *t*‐tests or a nonparametric alternative of the *t*‐test were performed.

##### BTT learning

We established different measures of learning, referring to performance during FBW conditions only. First, initial learning gain was calculated as the performance difference between the pre‐test and post‐test on the first day. Second, later learning gain was calculated as the performance difference between the post‐test on the first day and the post‐test on the last day. Finally, long‐term (or total) learning gain was calculated as the performance difference between the pre‐test on the first day and the post‐test on the last day. To investigate whether learning gains at each learning stage differed between groups, additional *t*‐tests or a nonparametric alternative of the *t*‐test were performed. To assess the associations among initial, later and long‐term learning gains, Pearson or Spearman correlation analyses were carried out between the learning measures in each group. Bonferroni correction was used to correct for the six comparisons (*p*_corr_ = *p*‐value × 6, alpha level set at *p*_*corr*_ = .05).

#### 2.5.2 MRS data analysis

To investigate whether baseline GABA+ levels were different between groups and across brain regions, we used a 4 × 2 (VOI × Group) two‐way mixed ANOVA in which VOI served as a within‐subject factor and Group served as a between‐subject factor. Since the Glx levels were not normally distributed, we used the nonparametric alternative of the two‐way mixed ANOVA.

#### 2.5.3 MRS‐BTT regression analysis

We used multiple linear regression analyses to investigate whether the baseline (resting state) levels of the neurometabolites (obtained on Day 1 prior to training) could predict the behavioral progress under FBW conditions in the combined groups as well as in each group separately. It has been recommended that, if an interaction between a continuous variable and another variable (continuous or categorical) is being tested in a regression analysis, the continuous variable(s) should be centered to avoid multicollinearity issues, which could result in inflated standard errors. Therefore, we used the mean‐centered neurometabolite levels in the multiple regression analyses in the combined groups.

## 3 RESULTS

### 3.1 Behavioral findings

#### 3.1.1 Behavioral data

**FIGURE 3:**
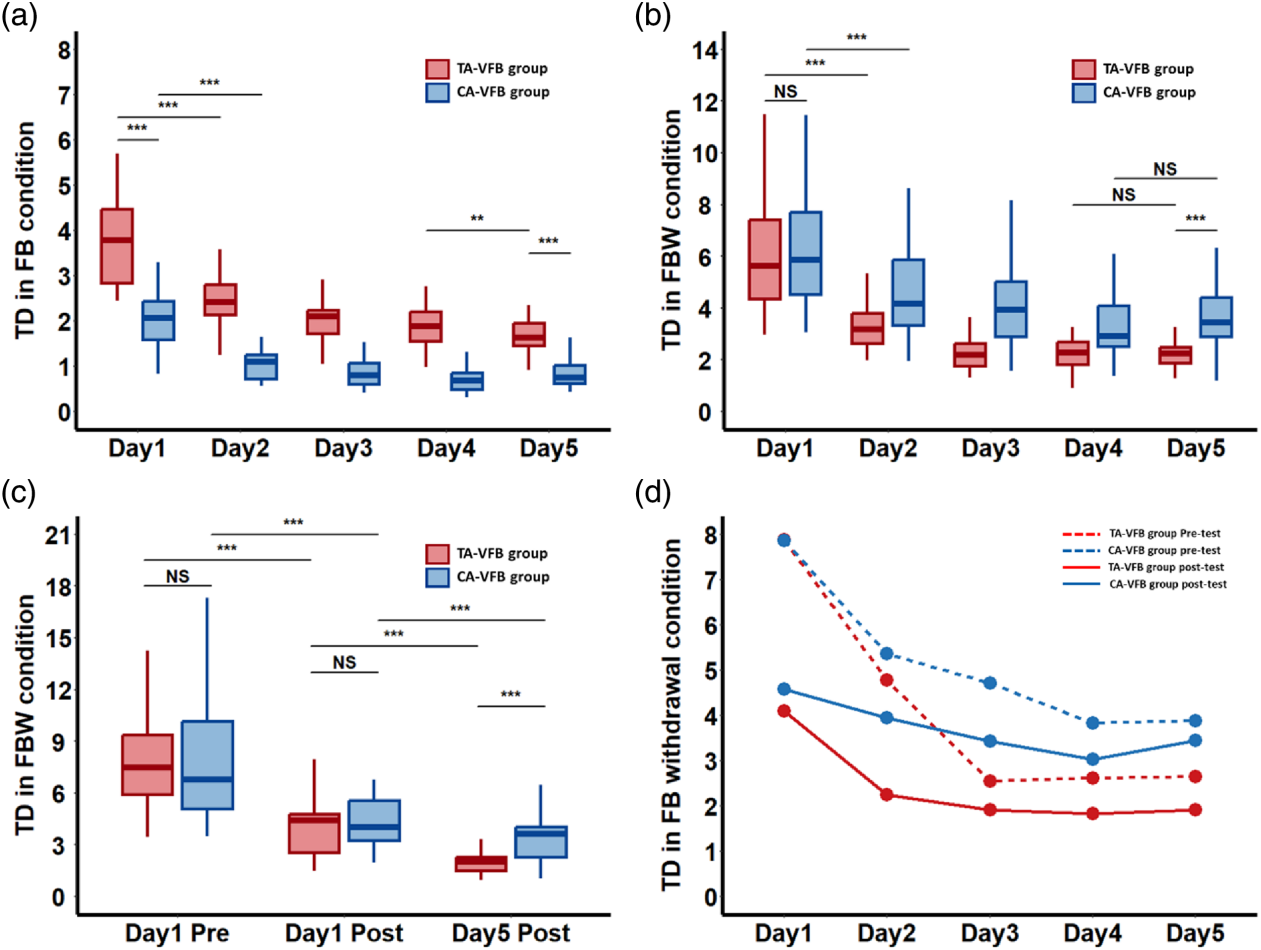
Behavioral data. (a) Boxplot of performance during the training (in the FB condition) across 5 days. (b) Boxplot of performance in the FBW condition across five training days. (c) Performance at Day 1 pre‐test, Day 1 post‐test and Day 5 post‐test. (d) Changes in the mean values of the pre‐tests and post‐tests across five training days in each group. CA‐VFB group, concurrent augmented visual feedback group; FB condition, feedback condition; FBW condition, feedback withdrawal condition; TA‐VFB group, terminal augmented visual feedback group; TD, tracking deviation.

Initial performance (i.e., Day 1 pre‐test) was not significantly different between the groups (*t* = 0.023, *p* = .98) (Figure 3c, Table 3), indicating that the groups started at the same level before training. Table 4 lists the mean and standard deviation of the behavioral performance of each group on different days. Table 5 lists results of the statistical analyses on the behavioral data obtained under the FB and FBW conditions.

**TABLE 3:** Results of statistical analyses comparing behavioral performance (obtained at pre‐tests and post‐tests) and learning gains between groups.

FBW condition	*n*	Mean	SD	Method	Statistic	*df*	*p*‐Value	Cohen's *d*
*Behavioral performance*								
Day 1 pre‐test in TA‐VFB group	26	7.89	2.9	Independent *t*‐test	*t* = 0.023	46.39	.982	0.006
Day 1 pre‐test in CA‐VFB group	25	7.87	3.54					
Day 1 post‐test in TA‐VFB group	26	4.10	1.88	Independent *t‐*test	*t* = − 0.879	48.22	.384	−0.246
Day 1 post‐test in CA‐VFB group	25	4.58	2.05					
Day 5 post‐test in TA‐VFB group	26	1.92	0.548	Wilcoxon rank‐sum test	*w* = 90	/	<.001***	0.620
Day 5 post‐test in CA‐VFB group	25	3.46	1.34					
*Learning gains*								
Initial learning gain in TA‐VFB group	26	3.79	2.33	Wilcoxon rank‐sum test	*w* = 386	/	.257	0.177
Initial learning gain in CA‐VFB group	25	3.28	3.29					
Later learning gain in TA‐VFB group	26	2.18	1.53	Independent *t*‐test	*t* = 2.45	38.94	.018*	0.687
Later learning gain in CA‐VFB group	25	1.12	1.53					
Long‐term learning gain in TA‐VFB group	26	5.97	2.64	Wilcoxon rank‐sum test	*w* = 439	/	.03*	0.526
Long‐term learning gain in CA‐VFB group	25	4.41	3.25					

Abbreviations: CA‐VFB group, concurrent augmented visual feedback group; *df*, degrees of freedom; FBW condition, feedback withdrawal condition; *n*, number of participants; SD, standard deviation; TA‐VFB group, terminal augmented visual feedback group.

* *p*‐Value <.05.

*** *p*‐Value <.001.

**TABLE 4:** Descriptive measures (mean ± standard deviation) of behavioral performance on each training day.

	TA‐VFB group			CA‐VFB group		
	FB condition	FBW condition		FB condition	FBW condition	
		Pre‐test	Post‐test		Pre‐test	Post‐test
Day 1	4.11 ± 1.68	7.89 ± 2.89	4.10 ± 1.88	2.06 ± 0.78	7.87 ± 3.54	4.58 ± 2.05
Day 2	2.51 ± 0.63	4.78 ± 2.18	2.26 ± 1.00	1.05 ± 0.33	5.36 ± 2.32	3.95 ± 1.67
Day 3	2.12 ± 0.58	2.55 ± 0.86	1.91 ± 0.60	0.86 ± 0.31	4.72 ± 2.11	3.43 ± 1.59
Day 4	1.95 ± 0.55	2.62 ± 0.98	1.83 ± 0.57	0.67 ± 0.24	3.84 ± 1.43	3.04 ± 1.44
Day 5	1.80 ± 0.57	2.64 ± 0.80	1.92 ± 0.55	0.84 ± 0.29	3.88 ± 1.58	3.46 ± 1.34

Abbreviations: CA‐VFB group, concurrent augmented visual feedback group; FB condition, feedback condition; FBW condition, feedback withdrawal condition; TA‐VFB group, terminal augmented visual feedback group.

**TABLE 5:** Results of the statistics on the behavioral data obtained during FB condition (training) and FBW condition (pre‐ and post‐tests).

Factor	Statistic	*df*	*p*‐Value
*FB condition*			
Group	151.72	1	<.001***
Day	164.05	2.29	<.001***
Group × day	9.12	2.29	<.001***
*FBW condition*			
Group	23.00	1	<.001***
Day	97.55	3.77	<.001***
Pre‐post	214.99	1	<.001***
Group × day	12.63	3.77	<.001***
Group × pre‐post	13.38	1	<.001***
Pre‐post × day	6.49	3.62	<.001***
Pre‐post × day × group	4.26	3.62	.002**

Abbreviations: *df*, degrees of freedom; FB condition, feedback condition; FBW condition, feedback withdrawal condition.

** *p*‐Value <.01.

*** *p*‐Value <.001.

##### FB‐supported performance condition

During the *FB condition* (*with FB manipulation*), the nonparametric alternative of the 2 × 5 (Group × Day) mixed ANOVA revealed a main effect of Day (*p* < .001), suggesting an overall improvement of performance over the course of training. Additionally, the significant main effect of Group (*p* < .001) indicated that the overall performance was better in the CA‐VFB group as compared with the TA‐VFB group (Figure 3a). This was anticipated because the CA‐VFB group could rely on the concurrent real‐time FB to steer performance online. Furthermore, the significant Group × Day (*p* < .001) interaction reflected that the performance of the CA‐VFB group was better than that of the TA‐VFB group at the beginning of training, but this difference became less pronounced toward the end of the training because the TA‐VFB group showed further improvement over the course of training (Figure 3a).

##### Performance under FBW condition as a test of learning

In the *FBW condition* (*absence of augmented FB*), the nonparametric alternative of the 2 × 2 × 5 (Group × Pre‐Post × Day) mixed ANOVA revealed a main effect of Day (*p* < .001), indicating an overall improvement of performance over the course of training (Figure 3b) and a main effect of Pre‐Post (*p* < .001), indicating that the overall performances were better at post‐test as compared with the pre‐test (Figure 3d). The interaction effect of Day × Pre‐Post (*p* < .001) was also significant, indicating that the performance improvement reduced over training days (Figure 3d). Additionally, a main effect of Group (*p* < .001) was observed, indicating that the overall performance of the TA‐VFB group was better than that of the CA‐VFB group during the FBW condition (Figure 3b). This is not surprising because the TA‐VFB group members were more familiar with such performance conditions during training, which better resembled the test conditions under FBW. Furthermore, we found a significant Group × Day interaction (*p* < .001), suggesting that the rate of performance improvement across training days was different between groups, that is, at the beginning there was a sharp progress in the TA‐VFB group, while the difference in performance improvement between groups got smaller with the relative greater progress in the CA‐VFB group in the later phase (Figure 3b,d). A Group × Pre‐Post interaction (*p* < .001) was also observed, indicating a greater performance improvement in the TA‐VFB group as compared to the CA‐VFB group after training. We also observed a significant interaction effect of Pre‐Post × Day × Group (*p* < .03) (Figure 3d), reflecting that the daily progress (i.e., the difference between pre‐test and post‐test) across training days differed between groups. More specifically, as compared with the CA‐VFB group, the TA‐VFB group showed greater daily progress during the first three training days but did not further improve performance during the last two training days. However, the CA‐VFB group showed slower but more continuous progress over all five training days. Results of the post‐hoc analyses comparing the behavioral performance of each group across five training days are reported in the supplementary Table S2.

#### 3.1.2 Performance and learning measures during different stages of learning

The statistics comparing behavioral performance at pre‐ and post‐tests and learning gains between groups are reported in Table 3. The initial learning outcome (i.e., Day 1 post‐test) was not significantly different between the groups (*t* = −0.879, *p* = .384) (Figure 3c), indicating that the groups ended at a similar level after initial training. The final learning outcome (i.e., Day 5 post‐test) was significantly different between the groups (*w* = 90, *p* < .001) (Figure 3c), indicating that the TA‐VFB group outperformed the CA‐VFB group after long‐term training. Additionally, the initial learning gain (difference between Day 1 pre‐test and post‐test) was not significantly different between groups (*w* = 386, *p* = .257). The later learning gain (*t* = 2.45, *p* = .018) and long‐term learning gain (*w* = 439, *p* = .03) were significantly larger in the TA‐VFB group as compared to the CA‐VFB group.

No correlations were found between the initial and later learning gains in each separate group: in CA‐VFB group (*r* = −.27, *p*_corr_ > .05) and in TA‐VFB group (*r* = −.15, *p*_corr_ >.05). However, significant correlations were found between the initial and long‐term learning gains in each separate group: in CA‐VFB group (*r* = .83, *p*_corr_ <.001) and in TA‐VFB group (*r* = .82, *p*_corr_ <.001). These results imply that the participants who made the most behavioral progress on the first training day also improved the most over long‐term training. Additionally, no correlations were found between the later and long‐term learning gains in each separate group: in CA‐VFB group (*r* = .18, *p*_corr_ >.05) and in TA‐VFB group (*r* = .39, *p*_corr_ >.05).

### 3.2 MRS results

#### 3.2.1 Differences in regional baseline neurometabolite levels

Table 6 lists mean and standard deviation of the baseline GABA+ and Glx levels in the four investigated MRS VOIs and also reports results of the 4 × 2 (VOI × Group) two‐way mixed ANOVA comparing the neurometabolite levels between the two groups and VOIs.

**TABLE 6:** Comparing tissue‐corrected baseline GABA+ and Glx levels between groups and VOIs.

	TA‐VFB group		CA‐VFB group		Effect	Statistic	*p*‐Value
	Mean	SD	Mean	SD			
*GABA+*							
M1	2.96	0.25	2.97	0.20			
DLPFC	2.61	0.20	2.68	0.28			
S1	2.96	0.17	2.98	0.27			
MT/V5	2.34	0.31	2.37	0.20			
					VOI	84.14	<.001***
					Group	1.219	.27
					VOI × group	0.163	.921
*Glx*							
M1	5.92	1.14	6.09	1.02			
DLPFC	7.16	0.85	7.12	0.91			
S1	6.32	1.11	6.69	1.20			
MT/V5	6.82	0.94	7.28	0.80			
					VOI	51.80	<.001***
					Group	1.111	.292
					VOI × group	2.384	.080

Abbreviations: CA‐VFB group, concurrent augmented visual feedback group; DLPFC, dorsolateral prefrontal cortex; GABA, gamma‐aminobutyric acid; Glx, glutamate and glutamine; M1, primary motor cortex; MT/V5, medial temporal visual cortex; S1, primary somatosensory cortex, TA‐VFB group, terminal augmented visual feedback group; VOI, voxel of interest.

*** *p*‐Value <.001.

##### GABA+ levels

**FIGURE 4:**
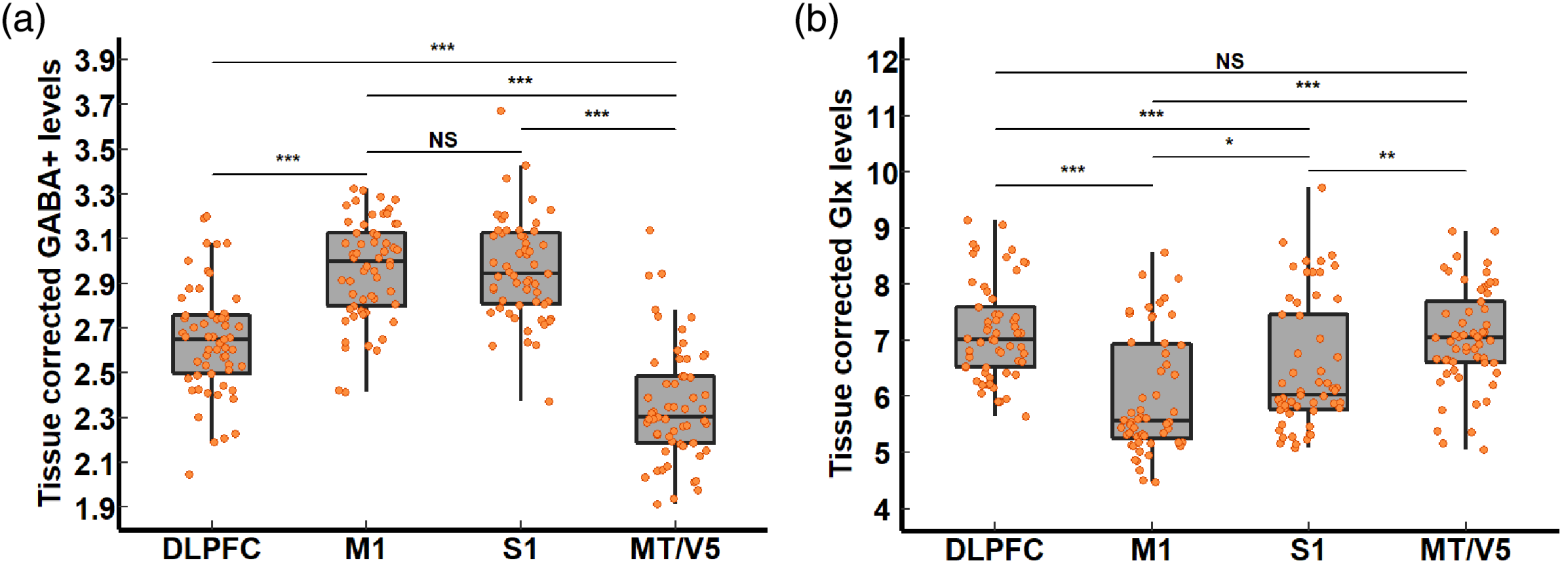
Regional differences in the MRS‐assessed baseline levels of (a) GABA+ and (b) Glx.

Results of the 4 × 2 (VOI × Group) two‐way mixed ANOVA revealed a significant main effect of VOI. Post‐hoc analyses indicated lower GABA+ levels in the DLPFC as compared to the M1 and S1, and lower GABA+ levels in the MT/V5 as compared to the other voxels of interest. However, there was no significant difference between GABA+ levels in the M1 and S1 (Figure 4a and supplementary Table S3). Furthermore, the main effect of Group was not significant, indicating that baseline GABA+ levels were not significantly different between groups. Additionally, the interaction effect of Voxel × Group was also not significant, indicating that the difference in the GABA+ levels between VOIs was not significantly different between groups (Table 6).

##### Glx levels

Results of the 4 × 2 (VOI × Group) nonparametric alternative of the two‐way ANOVA revealed a significant main effect of VOI. Post‐hoc analyses indicated lower Glx levels in the S1 as compared to the DLPFC and MT/V5, and lower Glx levels in the M1 as compared to the other voxels of interest. However, no significant difference was found between Glx levels in the DLPFC and MT/V5 (Figure 4b and supplementary Table S3). Furthermore, the main effect of Group was not significant, indicating that baseline Glx levels were not significantly different between groups. Additionally, the interaction effect of Voxel × Group was also not significant, indicating that the difference in the Glx levels between VOIs was not significantly different between groups (Table 6).

### 3.3 Predicting learning gains based on the baseline GABA+ levels

To investigate whether resting‐state GABA+ levels in the four VOIs could predict subsequent learning gains and whether the predictive value of baseline GABA+ levels on learning is dependent on the FB type, a series of multiple regression analyses were built with the following factors: (1) Group (TA‐VFB group, CA‐VFB group); (2) GABA+ levels in four VOIs (i.e., DLPFC, M1, S1, MT/V5); and (3) the interactive effects between GABA+ levels and Group.

#### 3.3.1 Initial learning gain

**FIGURE 5:**
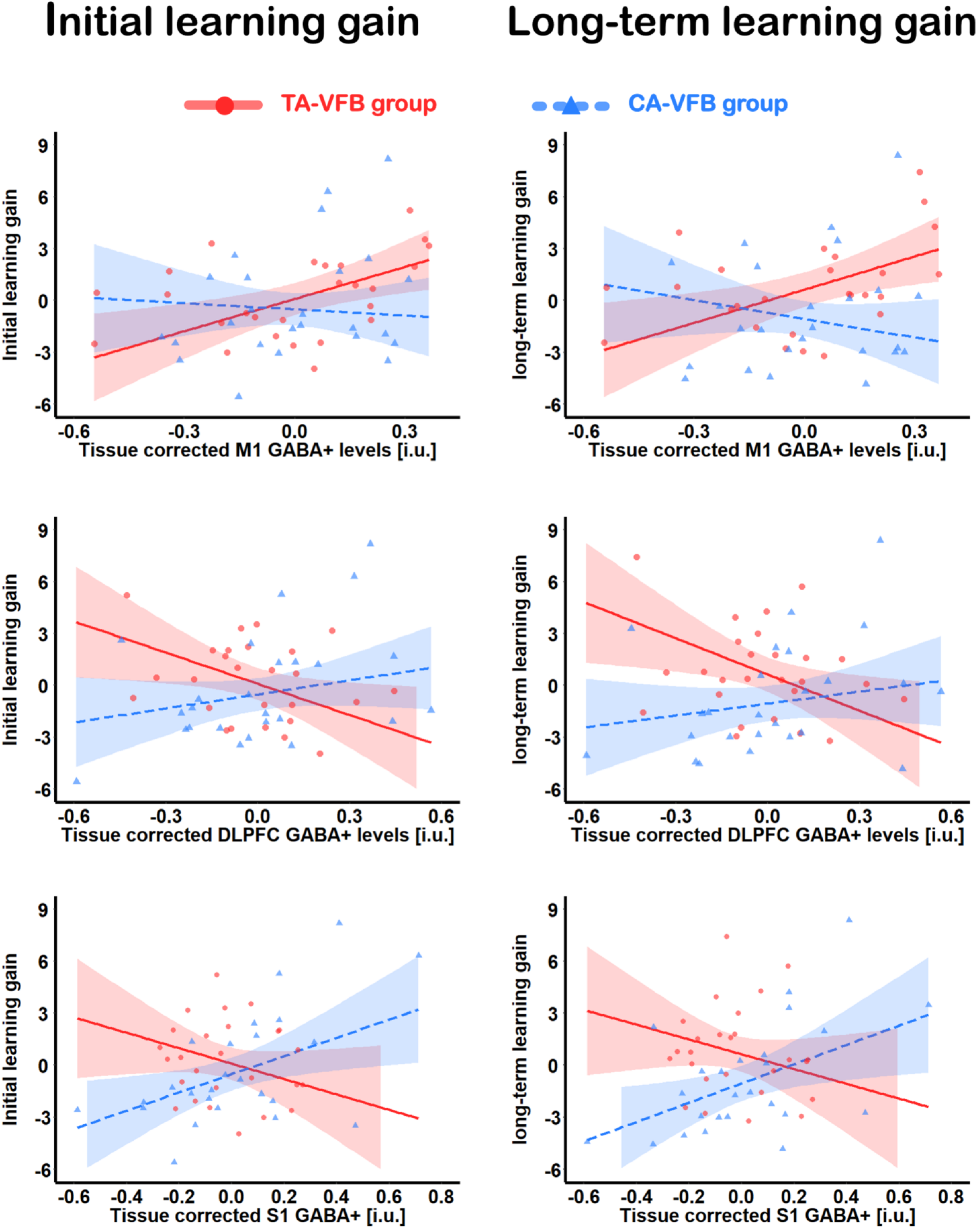
Interaction effects which were found to be significant in the multiple regression analyses.

**FIGURE 6:**
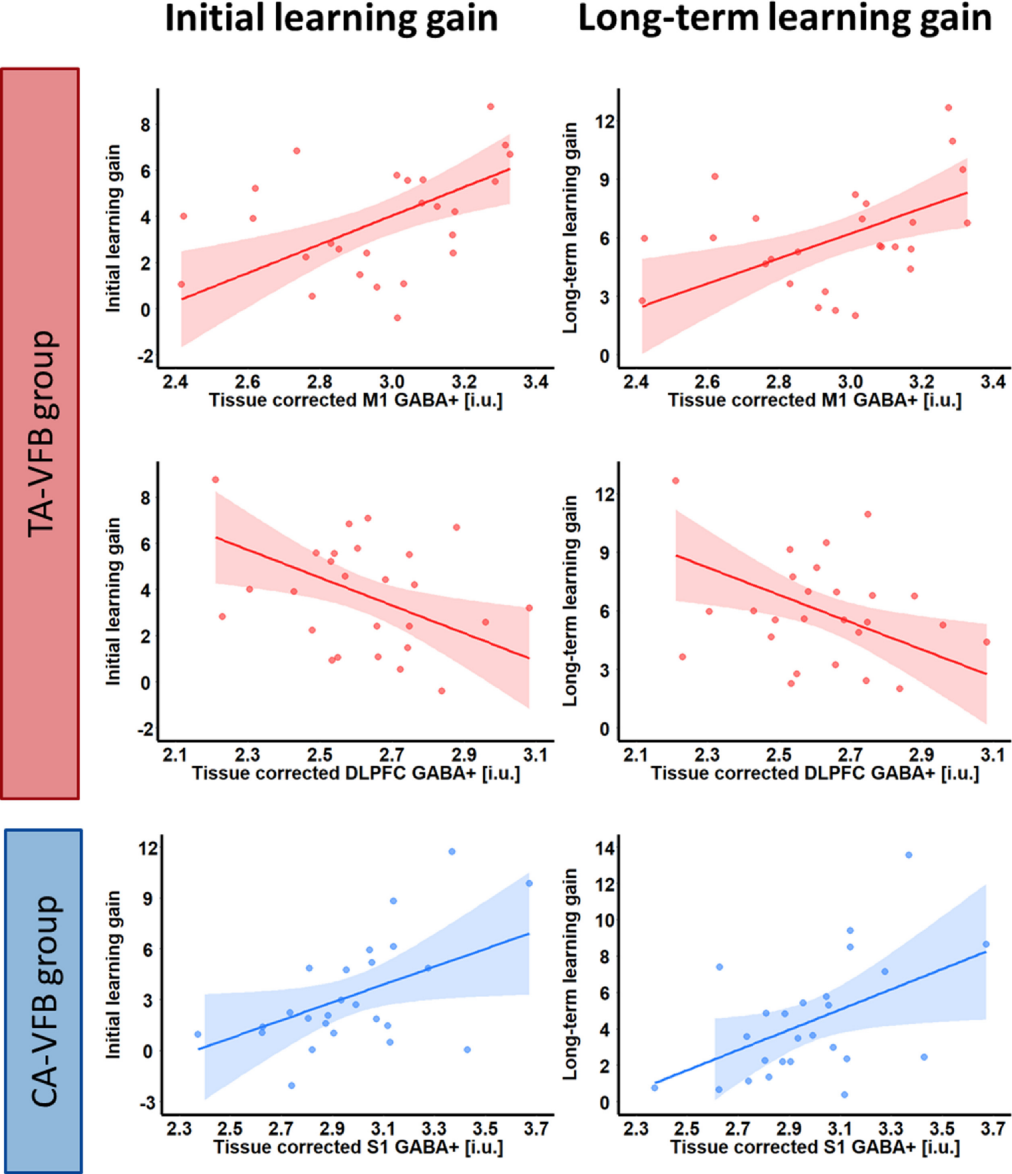
Tissue‐corrected GABA+ levels obtained from different MRS VOIs could significantly predict initial and long‐term learning gains in different feedback groups.

Table 7 summarizes the results of the multiple regression analysis predicting initial learning gain under the FBW condition. We observed that GABA+ levels in the M1 (*β* = .50, *t* = 2.99, *p* = .005) and the DLPFC (*β* = −.51, *t* = − 2.30, *p* = .027) independently contributed to predicting the initial learning gain in the TA‐VFB group. Furthermore, as shown by the interaction effects, there was a significant group difference with respect to the effect of GABA+ levels in the M1 (*p* = .038), DLPFC (*p* = .011), and S1 (*p* = .009) on predicting the initial learning gain. Figure 5 shows the significant interaction effects between neurometabolite levels and Group, obtained from the multiple regression analyses. Therefore, we further investigated the predictive value of baseline GABA+ levels in each group separately (Table 7). Results of the multiple regression analysis in the TA‐VFB group revealed that baseline GABA+ levels in the M1 positively predicted initial progress (*β* = .68, *t* = 3.64, *p* = .002), whereas GABA+ levels in the DLPFC negatively predicted initial progress (*β* = −.53, *t* = −2.80, *p* = .011). This suggested that higher GABA+ levels in the M1 were associated with higher initial learning gains while higher GABA+ levels in the DLPFC were associated with lower initial learning gains in the TA‐VFB group. Results of the multiple regression analysis in the CA‐VFB group showed that GABA+ levels in the S1 could positively predict initial learning gain in this group, suggesting that higher GABA+ levels in the S1 were associated with higher learning gains (*β* = .44, *t* = 2.20, *p* = .04). The associations between the GABA+ levels in different brain areas and initial learning gain are visualized in Figure 6 (left side).

**TABLE 7:** Results of the multiple linear regression models predicting initial learning gain using baseline GABA+ levels.

Group	*R* ^2^	*R* ^2^‐adj	*F*	Predictor	*β* (SE)	*B* (SE)	*t*	*p*‐Value
Combined groups	.45	.33	*F*_(9,41)_ = 3.76 *p* = .0016	Intercept	.03 (0.16)	0.09 (0.46)	0.20	.84
				M1‐GABA+	.50 (0.17)	6.20 (2.08)	2.99	.005**
				DLPFC‐GABA+0	−.51 (0.22)	−6.03 (2.62)	−2.30	.027*
				S1‐GABA+	−.36 (0.24)	−4.44 (2.90)	−1.53	.13
				MT/V5‐GABA+	.03 (0.15)	0.29 (1.62)	0.18	.86
				Group [CA‐VFB]	−.22 (0.23)	−0.61 (0.65)	−0.93	.36
				M1‐GABA+ × group [CA‐VFB]	−.60 (0.28)	−7.39 (3.45)	−2.15	.04*
				DLPFC‐GABA+ × group [CA‐VFB]	.74 (0.28)	8.74 (3.28)	2.73	.01*
				S1‐GABA+ × group [CA‐VFB]	.79 (0.29)	9.69 (3.55)	2.66	.01**
				MT/V5‐GABA+ × group [CA‐VFB]	.42 (0.29)	4.56 (3.00)	1.52	.14
TA‐VFB group	.44	.33	*F*_(4,21)_ = 4.18 *p* = .012	Intercept	.00 (0.16)	13.62 (8.07)	1.69	.11
				M1‐GABA+	.68 (0.19)	6.20 (1.70)	3.64	.002**
				DLPFC‐GABA+	−.53 (0.19)	−6.03 (2.15)	−2.80	.01*
				S1‐GABA+	−.33 (0.17)	−4.44 (2.37)	−1.87	.08
				MT/V5‐GABA+	.04 (0.18)	0.29 (1.33)	0.22	.83
CA‐VFB group	.45	.34	*F*_(4,20)_ = 4.10* *p* = .014	Intercept	.00 (0.16)	−27.54 (9.78)	−2.81	.01*
				M1‐GABA+	−.07 (0.20)	−1.19 (3.19)	−0.37	.71
				DLPFC‐GABA+	.23 (0.19)	2.72 (2.29)	1.18	.25
				S1‐GABA+	.44 (0.20)	5.25 (2.39)	2.20	.04*
				MT/V5‐GABA+	.29 (0.17)	4.84 (2.92)	1.66	.11

Abbreviations: *B*, regression coefficient; CA‐VFB group, concurrent augmented visual feedback group; DLPFC, dorsolateral prefrontal cortex; M1, primary motor cortex; MT/V5, medial temporal visual cortex; *R* ^2^‐adj, adjusted *R* ^2^; S1, primary somatosensory cortex; SE, standard error; TA‐VFB group, terminal augmented visual feedback group; *β*, standardized regression coefficient.

* *p*‐Value ≤0.05.

** *p*‐Value <.01.

#### 3.3.2 Later learning gain

The multiple regression analysis predicting later learning gain (under the FBW condition) is summarized in Supplementary Table S4. These results indicated that later learning gain was not significantly predicted by the proposed factors (*F*(9,41) = 1.128, *p* > .05).

#### 3.3.3 Long‐term learning gain

Table 8 summarizes the results of the multiple regression analysis predicting long‐term learning gain. Moreover, this model suggested that GABA+ levels in the M1 and DLPFC voxels contributed independently to long‐term learning gain (*p* = .007 and *p* = .017, respectively) in the TA‐VFB group. Furthermore, as shown by the interaction effects, there was a significant group difference with respect to the effect of GABA+ levels in the M1 (*p* = .01), DLPFC (*p* = .012), and S1 (*p* = .015) on long‐term learning gain. Figure 5 shows the significant interaction effects between neurometabolite levels and Group, obtained from the multiple regression analyses. Therefore, we further investigated the predictive value of baseline GABA+ levels in these voxels in each group separately (Table 8). Results of the multiple regression analysis in the TA‐VFB group revealed that baseline GABA+ levels in M1 positively predicted long‐term learning (*β* = .62, *t* = 3.21, *p* = .0042), whereas GABA+ levels in DLPFC negatively predicted long‐term learning (*β* = −.54, *t* = − 2.78, *p* = .011). Results of the multiple regression analysis in the CA‐VFB group showed that GABA+ levels in the S1 positively predicted long‐term learning (*β* = .48, *t* = 2.26, *p* = .036). The associations between the GABA+ levels in different brain areas and long‐term learning gain are visualized in Figure 6 (right side). The findings obtained for initial and overall long‐term learning gain show a converging pattern.

**TABLE 8:** Results of the multiple linear regression models predicting long‐term learning gain using baseline GABA+ levels.

Group	*R* ^2^	*R* ^2^‐adj	F	Predictor	*β* (SE)	*B* (SE)	*t*	*p*‐Value
Combined groups	.44	.32	*F*_(9,41)_ = 3.66 *p* = .002	Intercept	.21 (0.16)	0.63 (0.50)	1.27	.21
				M1‐GABA+	.48 (0.17)	6.40 (2.25)	2.85	.007**
				DLPFC‐GABA+	−.56 (0.23)	−6.99 (2.83)	−2.47	.018*
				S1‐GABA+	−.32 (0.24)	−4.27 (3.13)	−1.36	.18
				MT/V5‐GABA+	.16 (0.15)	1.86 (1.76)	1.06	.29
				Group [CA‐VFB]	−.56 (0.23)	−1.68 (0.71)	−2.38	.022*
				M1‐GABA+ × group [CA‐VFB]	−.76 (0.28)	−10.05 (3.73)	−2.70	.01*
				DLPFC‐GABA+ × group [CA‐VFB]	.74 (0.28)	9.30 (3.55)	2.62	.012*
				S1‐GABA+ × group [CA‐VFB]	.74 (0.29)	9.82 (3.85)	2.55	.014*
				MT/V5‐GABA+ × group [CA‐VFB]	.27 (0.28)	3.11 (3.25)	0.96	.34
TA‐VFB group	.41	.98	*F*_(4,21)_ = 3.65 *p* = .020	Intercept	.00 (0.16)	13.54 (9.46)	1.54	.17
				M1‐GABA+	.62 (0.19)	6.41 (2.00)	3.21	.004**
				DLPFC‐GABA+	−.54 (0.20)	−6.99 (2.52)	−2.78	.011*
				S1‐GABA+	−.27 (0.18)	−4.27 (2.78)	−1.53	.140
				MT/V5‐GABA+	.22 (0.19)	1.86 (1.56)	1.20	.24
CA‐VFB group	.40	.28	*F*_(4,20)_ = 3.36 *p* = .029	Intercept	.00 (0.17)	−19.2 (10.10)	−1.91	.07
				M1‐GABA+	−2.27 (0.21)	−3.64 (3.29)	−1.11	.28
				DLPFC‐GABA+	−.20 (0.20)	−2.31 (2.37)	0.98	.34
				S1‐GABA+	.48 (0.21)	5.55 (2.46)	2.26	.035*
				MT/V5‐GABA+	.30 (0.18)	4.97 (3.02)	1.65	.11

Abbreviations: *B*, regression coefficient; CA‐VFB group, concurrent augmented visual feedback group; DLPFC, dorsolateral prefrontal cortex; M1, primary motor cortex; MT/V5, medial temporal visual cortex; *R* ^2^‐adj, adjusted *R* ^2^; S1, primary somatosensory cortex; SE, standard error; TA‐VFB group, terminal augmented visual feedback group; *β*, standardized regression coefficient.

* *p*‐Value ≤.05.

** *p*‐Value <.01.

### 3.4 Predicting learning gains based on baseline Glx levels

To investigate whether baseline Glx levels in the four VOIs predicted learning gains and whether this depended on the FB type, a series of multiple regression analyses were built including the following factors: (1) Group (TA‐VFB group, CA‐VFB group); (2) Glx levels in four VOIs (i.e., DLPFC, M1, S1, MT/V5); and (3) the interactive effects between Glx levels and Group.

#### 3.4.1 Initial learning gain

The multiple regression analysis indicated that initial learning gain could not be significantly predicted by the proposed factors (*F*(9,40) = 1.518, *p* > .05) (Table 9).

**TABLE 9:** Results of the multiple linear regression models predicting initial learning gain using baseline Glx levels.

Group	*R* ^2^	*R* ^2^‐adj	*F*	Predictor	*B* (SE)	*t*	*p*‐Value
Combined groups	.25	.087	*F*_(9,40)_ = 1.52 *p* = .17	intercept	0.07 (0.58)	0.12	.90
				M1‐Glx	−0.76 (0.99)	−0.77	.45
				DLPFC‐Glx	1.65 (0.91)	1.81	.08
				S1‐Glx	0.51 (1.09)	0.46	.65
				MT/V5‐Glx	−0.62 (0.75)	−0.83	.41
				Group [CA‐VFB]	−0.17 (0.84)	−0.20	.84
				M1‐Glx × group [CA‐VFB]	−1.88 (1.63)	−1.16	.25
				DLPFC‐Glx × group [CA‐VFB]	−0.32 (1.42)	−0.23	.82
				S1‐Glx × group [CA‐VFB]	−0.97 (1.42)	−0.69	.50
				MT/V5‐Glx × group [CA‐VFB]	1.88 (1.34)	1.41	.17

Abbreviations: B, regression coefficient; CA‐VFB group, concurrent augmented visual feedback group; DLPFC, dorsolateral prefrontal cortex; M1, primary motor cortex; MT/V5, medial temporal visual cortex; *R* ^2^‐adj, adjusted *R* ^2^; S1, primary somatosensory cortex; SE, standard error.

#### 3.4.2 Later learning gain

The multiple regression analysis revealed that later learning gain could not be significantly predicted by the proposed factors (*F*(9,40) = 1.524, *p* > .05) (supplementary Table S5).

#### 3.4.3 Long‐term learning gain

The multiple regression analysis revealed that overall long‐term learning gain could not be significantly predicted by the proposed factors (*F*(9,40) = 1.268, *p* > .05) (Table 10).

**TABLE 10:** Results of the multiple linear regression models predicting long‐term learning gain using baseline Glx levels.

Group	*R* ^2^	*R* ^2^‐adj	*F*	Predictor	*B* (SE)	*t*	*p*‐Value
Combined groups	.222	.047	*F*_(9,40)_ = 1.268 *p* = .284	Intercept	0.63 (0.64)	0.99	.33
				M1‐Glx	−0.87 (1.09)	−0.80	.43
				DLPFC‐Glx	1.90 (1.00)	1.89	.07
				S1‐Glx	0.58 (1.20)	0.48	.63
				MT/V5‐Glx	−0.57 (0.83)	−0.69	.49
				Group [CA‐VFB]	−1.17 (0.93)	1.29	.20
				M1‐Glx × group [CA‐VFB]	−0.85 (1.79)	−0.47	.64
				DLPFC‐Glx × group [CA‐VFB]	−0.89 (1.56)	−0.57	.57
				S1‐Glx × group [CA‐VFB]	−0.51 (1.56)	−0.33	.75
				MT/V5‐Glx × group [CA‐VFB]	0.47 (1.48)	0.31	.75

Abbreviations: B, regression coefficient; CA‐VFB group, concurrent augmented visual feedback group; DLPFC, dorsolateral prefrontal cortex; M1, primary motor cortex; MT/V5, medial temporal visual cortex; *R* ^2^‐adj, adjusted *R* ^2^; S1, primary somatosensory cortex; SE, standard error.

## 4 DISCUSSION

MRS measures of neurometabolite levels were obtained to investigate the relationship between baseline levels of GABA+ and Glx in four motor learning‐related brain areas and the behavioral progress made at different stages of motor learning. Over the course of 5 days, participants were trained on a bimanual task and received augmented VFB either during the execution of the task (CA‐VFB group) or after the completion of the trial (TA‐VFB group). At the behavioral level, participants who were trained with after‐trial visual FB (i.e., TA‐VFB group) outperformed those who were trained with online visual FB (i.e., CA‐VFB group) when assessing learning (FBW). At the neurochemical‐behavioral level, initial and long‐term motor learning progress was positively predicted by GABA+ levels in the M1 but negatively predicted by GABA+ levels in the DLPFC in the TA‐VFB group. In the CA‐VFB group, however, learning was positively predicted by GABA+ levels in the S1. Glx levels did not significantly predict the behavioral progress at any stage.

### 4.1 FB and motor learning

Motor performance in the CA‐VFB group, receiving online visual FB, outperformed the TA‐VFB group when augmented FB was available during training (the *FB condition*). In contrast, performance in the TA‐VFB group, only receiving visual FB after the end of each trial during training, outperformed the CA‐VFB group when weaned from augmented FB (*FBW condition*, *during pre‐ and post‐tests*). The latter condition served to assess learning progress. Consequently, whereas receiving concurrent/online FB boosted performance during the training, it hampered learning progress due to overreliance on concurrent FB. On the contrary, although participants of the TA‐VFB group faced a greater challenge during training because no concurrent FB was made available to them, they ultimately learned the skill better than in the CA‐VFB group. The TA‐VFB group was better prepared to FB‐deprival conditions because they likely developed a more advanced internal error evaluation and correction strategy based on somatosensory input (Schmidt & Wulf, 1997; Vander Linden et al., 1993; Winstein et al., 1996). These findings support the “guidance hypothesis of information FB,” which suggests a supportive and guiding role of FB on performance as long as it is present, but a detrimental role that becomes apparent under FBW conditions (Salmoni et al., 1984; Schmidt, 1991). Numerous studies have manipulated different properties of FB (such as their timing and frequency) to assess their effect on motor learning (for reviews, see Newell, 1976 and Swinnen, 1996). Studies have also reported that concurrent, as compared with terminal, FB can impair learning (Ranganathan & Newell, 2009; Schmidt & Wulf, 1997; Swinnen et al., 1990). Concurrent FB makes performers increasingly dependent on the strong guidance provided by the FB, thus hampering internal error evaluation based on proprioceptive input. This dependence is reduced when terminal FB is provided (for a review, see Schmidt, 1991). This should be considered when designing training protocols to maximize the learning outcome.

### 4.2 Regional specificity of MRS‐measured levels of GABA+ and Glx

Our results demonstrated that concentrations of GABA+ and Glx varied across different VOIs. For GABA+, we observed the highest levels in the M1 and S1, followed by the DLPFC and MT/V5. For Glx, the highest levels were measured in the DLPFC and MT/V5, followed by the S1 and then the M1. This is consistent with converging evidence that GABA+ and Glx concentrations are not homogenously distributed across brain regions (Grachev & Apkarian, 2000a, 2000b; Maes et al., 2021; Rodríguez‐Nieto et al., 2023). That GABA+ levels in the DLPFC are lower than in the S1 and M1 area is consistent with a previous study (Maes et al., 2021). Additionally, previous MRS studies have provided support for an anterior–posterior gradient in GABA+ levels, with greater GABA+ levels in the posterior regions (Chalavi et al., 2018; Hermans et al., 2018; Maes et al., 2018; Mikkelsen et al., 2018; Porges et al., 2017; Takei et al., 2016) even though inconsistent results have also been reported. For example, some studies reported no significant differences in GABA levels between the frontal and occipital cortex (Hermans et al., 2018; Marsman et al., 2017) or even higher GABA+ levels in the frontal as compared to the parietal cortex (Gao et al., 2013). Moreover, we investigated for the first time GABA+ levels in the MT/V5 (at the conjunction of the parietal and occipital cortex) and showed that these were lower than those in the S1/M1 area and DLPFC.

With respect to Glx, we showed lower levels in the DLPFC as compared to the S1 and M1 area, consistent with previous research (Grachev & Apkarian, 2000a). Moreover, a study showed no significant difference in Glx levels between the frontal and the posterior midline voxels (Gao et al., 2013). Our results could not establish a significant difference in Glx levels between the DLPFC and MT/V5.

### 4.3 Baseline levels of GABA+, not Glx, predict behavioral progress

The brain–behavior analyses did not reveal any associations between Glx levels and learning. However, we did observe that initial and long‐term motor learning progress, as indexed by performance under FBW conditions, were positively predicted by GABA+ levels in the M1 and negatively predicted by GABA+ levels in the DLPFC in the TA‐VFB group using terminal visual FB during training. Conversely, motor learning progress was positively predicted by GABA+ levels in the S1 area in participants of the CA‐VFB group who received concurrent visual FB during training. These findings appear only partially consistent with the proposed hypotheses, as discussed next.

Positive associations between baseline M1 GABA+ levels and performance have been demonstrated in previous studies for various types of sensorimotor tasks (Cassady et al., 2019; Chalavi et al., 2018; Stagg et al., 2011). Less evidence is available for positive associations between baseline M1 GABA+ levels and learning. Here, we observed that participants with higher M1 GABA+ levels were more successful in acquiring the bimanual skill during the initial short‐term learning stage as well as during the longer term. We tentatively suggest that the higher M1 GABA+ levels may be linked to better construction of distinct memory representations in the M1 for the different subtasks. However, while we expected this association to be present in both groups, it was only observed in the TA‐VFB group. While the TA‐VFB group experienced more difficulties improving motor performance during training as compared to the CA‐VFB group (using concurrent visual FB), they performed better than the CA‐VFB group when weaned from augmented FB during tests of learning (FBW conditions). This may have promoted the building of more advanced motor memory representations in M1 in the TA‐VFB group.

Positive associations between baseline GABA+ levels and learning have also been reported using other paradigms. Heba et al. measured perceptual improvements by comparing tactile sensitivity of the index finger before and after repetitive somatosensory stimulation on the right hand (pre‐ and post‐test measurements). They showed that both the tactile sensitivity learning gains and final learning outcomes were positively associated with baseline SM1 GABA+ levels (Heba et al., 2016). It has been proposed that the role of higher baseline GABA+ levels in better sensorimotor performance and enhanced retrieval of memorized information may be mediated by suppressing the interference induced by irrelevant stimuli (Li et al., 2022). Altogether, we speculate that in the TA‐VFB group, higher baseline M1 GABA+ levels promoted/facilitated building better memory representations in the motor cortex, and this could have been mediated by suppressing the interference induced by irrelevant stimuli (Heba et al., 2016; Li et al., 2022).

Conversely, with respect to neurochemical dynamics, lowering GABA+ levels may promote plasticity by a release from inhibition and facilitation of neural interactions even though this typically refers to experimentally‐induced modulation of GABA+ levels in the SM1 to facilitate learning (Stagg et al., 2011). Other studies looking into the dynamics of SM1 GABA+ changes during task learning have observed a decrease in the MRS‐assessed GABA+ levels during short‐term (Chalavi et al., 2018; Floyer‐Lea et al., 2006; Kolasinski et al., 2019; Maes et al., 2021; Nettekoven et al., 2022) and long‐term motor learning (Sampaio‐Baptista et al., 2015), which may be consistent with a release from inhibition to promote motor learning. Furthermore, a study in older participants showed that participants with higher baseline GABA+ levels were more likely to exhibit a greater decrease in GABA+ levels during motor sequence training, which was linked to greater motor learning magnitude (King et al., 2020). From this perspective, higher resting‐state GABA+ levels in task‐related brain areas (as we reported here) may embed a larger window for training‐induced modulation to obtain a release from inhibition via a reduction of GABA to induce plasticity and learning.

The observed negative association between DLPFC GABA+ levels and motor learning progress was not consistent with our preliminary hypothesis. Interestingly, this was only observed in participants of the TA‐VFB group. While we anticipated higher DLPFC GABA+ levels to support the building of distinct memory representations for the sub‐movements in M1, this was associated with lower progress during short‐ and long‐term learning. Positive associations between DLPFC GABA+ levels and performance/learning have been shown for various types of tasks in previous studies, but negative associations have been less prominently reported. As part of the prefrontal cortex, DLPFC plays a converging role between the inputs from the sensory processing areas and the outputs to the motor areas. It has also been implicated in numerous higher cognitive functions, such as task switching, planning, and attention control (Sakagami et al., 2006; Yoon et al., 2016). However, it is important to consider that the association between GABA+ levels and behavior might be contingent upon the particular function that is performed by the DLPFC in the different tasks under investigation. Thus far, to the best of our knowledge, no study has investigated the association between neurometabolite levels in the DLPFC and motor learning. However, Scholl et al. (2017) investigated the relationship between baseline GABA+ levels in the dorsal anterior cingulate cortex (dACC) and reward‐guided learning. Results revealed that lower baseline GABA+ levels in the dACC were associated with better reward‐guided learning (Scholl et al., 2017). It is important to note that skill learning may be partially distinct from reward learning in terms of underlying brain regions. Nevertheless, it is possible to conceive that lower baseline DLPFC levels may be associated with the more flexible exploration of the task space that is composed of different subtasks. In the present study, practice in the absence of concurrent FB may create more optimal conditions for active exploration of the task space. As such, lower GABA+ levels may promote flexible task space exploration while acquiring different task variants (Li et al., 2022).

Finally, we hypothesized an association between baseline GABA+ levels in sensory‐processing regions and motor learning as a function of the sensory source that was prominently available to the participants during task training. Specifically, we anticipated that GABA+ levels in the MT/V5 would be associated with the amount of learning in the CA‐VFB group because participants of the latter group had access to real‐time augmented VFB during task training (vision‐based practice, externally‐guided). Conversely, a positive association between GABA+ levels in S1 and learning was expected in the TA‐VFB group because the absence of online visual FB forced participants to process the somatosensory consequences associated with movement production (proprioception‐based practice, internally‐guided). Please note that somatosensory input was equally available in the CA‐VFB group but its processing may have been suppressed as a result of overreliance on abundant online visual FB.

Surprisingly, GABA+ levels in the S1 brain region were associated with learning gains in the CA‐VFB, but not the TA‐VFB group. This result can possibly be accounted for by focusing on the shift from performance in the presence of concurrent FB to FB removal when somatosensory input becomes more critical. Thus, a viable explanation is that those participants with higher baseline S1 GABA+ levels were better able to cope with this shift to non‐visually‐supported performance which requires extra processing of somatosensory information. Nevertheless, future research is required to confirm this hypothesis. Furthermore, it is clear that other brain areas can be considered in comparing internally‐guided versus externally‐guided movement performance conditions because the networks involved in these two types of control are clearly different (Debaere et al., 2003; Swinnen & Wenderoth, 2004). Previous work has also shown that learners who shift from visually‐supported to nonvisual performance conditions show temporary preservation of brain activity in MT/V5 even though the visual input is no longer available (Ronsse et al., 2011). This underscores the dominance of visual information processing, even in the absence of actual visual input, when weaning from visual input is required to shift to proprioception‐based performance (during the FBW condition). This was challenging as can be inferred from the CA‐VFB group's lower performance levels under the FBW conditions.

One question that might arise here is why GABA+ levels from different brain regions could predict learning gains in the two experimental groups, that is, the S1 GABA+ levels in the CA‐VFB group and the M1 GABA+ levels in the TA‐VFB group. Considering that the S1‐M1 overlap rate was not significantly different between the groups and given that the overlap rate between the S1 and M1 voxels was less than 50% in both groups, we hypothesize that GABA+ levels in the non‐overlapping parts (i.e., the more frontal, motor‐related cortex for the M1 voxel and the more parietal, somatosensory processing‐related cortex for the S1 voxel) are possibly responsible for the differential associations observed between the learning gain and the GABA+ levels obtained from the M1 and S1 regions in each group.

Taken together, whether higher or lower baseline GABA+ levels benefit performance or learning might depend on the function of the considered brain area in the execution of a specific task and the environmental training context. Moreover, in accounting for neurometabolite‐behavioral associations, GABA appears a more promising candidate than Glx which did not reveal any associations with learning capability.

### 4.4 Limitations

We observed positive associations between higher baseline GABA+ levels and initial/long‐term learning gain (the S1 area in CA‐VFB group and the M1 area in TA‐VFB group) as well as a negative association between baseline GABA+ levels in DLPFC and initial/long‐term learning gain in TA‐VFB group, suggesting an important role of baseline GABA+ levels in motor learning. Despite the new information provided by this study, some limitations need to be considered. First, besides GABA, Glu has been reported to be associated with human learning. However, based on the results we obtained under 3 T MR, we did not observe any significant correlation between the Glx levels and motor learning. Given that the Glx concentrations, measured under 3 T MR scanner, contain a large amount of Glutamine, disentangling the contribution of Glu in learning becomes challenging. Therefore, further studies investigating associations between neurometabolites and human learning are warranted. Second, establishing associations between GABA+ levels and motor learning is only a first step in exploring whether baseline neurometabolite levels predict learning. A better mechanistic understanding of underlying processes is required to push the envelope of causality.

## 5 CONCLUSION

Levels of neurometabolites obtained during rest predict future progress with learning a motor skill across the short and longer term. The conditions under which motor tasks are trained partially determine which brain regions are relevant candidates for predicting learning. Specifically, GABA+ levels in the primary motor cortex (M1) showed a positive and GABA+ levels in the DLPFC showed a negative association with learning capacity under internally‐guided practice regimes in which proprioceptive information was prominently used. Under externally‐guided training regimes with real‐time augmented VFB provision, GABA+ levels in the primary somatosensory cortex (S1) were a dominant predictor of learning gains. These findings highlight the potential role of baseline GABA+ levels obtained from different task‐related brain areas in predicting initial and long‐term motor learning gains. As such, baseline GABA+ constitutes a potential biomarker for motor learning capacity in young adults.

## AUTHOR CONTRIBUTIONS

Hong Li: Designed research, conducted research, analyzed data, wrote and revised manuscript. Sima Chalavi: Designed research, wrote and revised manuscript. Amirhossein Rasooli: Conducted research and revised manuscript. Geraldine Rodríguez Nieto: Revised manuscript. Caroline Seer: Revised manuscript. Mark Mikkelsen: Supported data analysis and revised manuscript. Richard A. E. Edden: Revised manuscript and supported data analysis. Dante Mantini: Revised manuscript. Stefan Sunaert: Secured operation of the MRI research equipment. Ron Peeters: Secured operation of the MRI data collection. Stephan P. Swinnen: Secured funding, designed research, wrote and revised manuscript.

## CONFLICT OF INTEREST STATEMENT

The authors declare no competing financial interests.

## Supporting information


**TABLE S1.** MRS quality measures
**TABLE S2.** Results of the post‐hoc analyses on the behavioral data comparing performance between training days.
**TABLE S3.** Results of the post‐hoc analyses, comparing MRS‐assessed levels of GABA+ and Glx between different brain regions.
**TABLE S4.** Results of the multiple linear regression model predicting later learning gain using baseline levels of GABA+.
**TABLE S5.** Results of the multiple linear regression model predicting later learning gain using baseline levels of Glx.
**FIGURE S1.** Heatmap of the locations of MRS VOIs and the MRS spectra obtained from these VOIs in the two groups.
**FIGURE S2.** Overlap between the M1 and S1 voxels in each separate group.

## Data Availability

The data that support the findings of this study are available on request from the corresponding author. The data are not publicly available due to privacy or ethical restrictions.
