# Supplementary material for: Baseline GABA+ levels in areas associated with sensorimotor control predict initial and long‐term motor learning progress
Source: Hum Brain Mapp. 2023 Dec 22;45(1):e26537. doi: 10.1002/hbm.26537 (PMC10789216; doi:10.1002/hbm.26537)
Supplement: Supplementary file 1 — TABLE S1. MRS quality measures TABLE S2. Results of the post‐hoc analyses on the behavioral data comparing performance between training days. TABLE S3. Results of the post‐hoc analyses, comparing MRS‐assessed levels of GABA+ and Glx between different brain regions. TABLE S4. Results of the multiple linear regression model predicting later learning gain using baseline levels of GABA+. TABLE S5. Results of the multiple linear regression model predicting later learning gain using baseline levels of Glx. FIGURE S1. Heatmap of the locations of MRS VOIs and the MRS spectra obtained from these VOIs in the two groups. FIGURE S2. Overlap between the M1 and S1 voxels in each separate group. [file HBM-45-e26537-s001.docx]

# Supplementary materials

**Table S1** MRS quality measures

| Voxel | Measure | TA-VFB group | CA-VFB group | Statistic | *p*-value |
| --- | --- | --- | --- | --- | --- |
| M1 | *GABA* |  |  |  |  |
|  | SNR | 19.44 (3.16) | 20.93 (3.60) | t = -1.6 | 0.12 |
|  | FWHM | 18.24 (1.12) | 18.37 (0.96) | t = -0.46 | 0.65 |
|  | Fit error | 3.63 (0.71) | 3.63 (0.81) | t = 0.02 | 0.98 |
|  | *Glx* |  |  |  |  |
|  | SNR | 15.06 (4.26) | 17.02 (3.65) | t = -1.76 | 0.08 |
|  | FWHM | 15.12 (7.01) | 13.84 (1.15) | w =329.5 | 0.94 |
|  | Fit error | 4.86 (1.21) | 4.56 (1.45) | w =397 | 0.18 |
| DLPFC | *GABA* |  |  |  |  |
|  | SNR | 18.34 (3.50) | 17.94 (3.30) | t = 0.41 | 0.68 |
|  | FWHM | 18.68 (1.11) | 19.00 (1.63) | t = -0.82 | 0.41 |
|  | Fit error | 4.25 (1.23) | 4.14 (0.98) | w = 334 | 0.87 |
|  | *Glx* |  |  |  |  |
|  | SNR | 18.37 (3.54) | 18.05 (4.12) | t = 0.30 | 0.77 |
|  | FWHM | 14.88 (1.36) | 15.08 (1.37) | w = 293 | 0.56 |
|  | Fit error | 4.20 (0.96) | 4.13 (0.96) | t = 0.25 | 0.81 |
| S1 | *GABA* |  |  |  |  |
|  | SNR | 22.00 (3.88) | 22.27 (4.96) | t = -0.22 | 0.83 |
|  | FWHM | 18.18 (0.98) | 18.58 (1.06) | w = 259.5 | 0.22 |
|  | Fit error | 3.53 (0.78) | 3.62 (0.48) | w = 257.5 | 0.21 |
|  | *Glx* |  |  |  |  |
|  | SNR | 18.57 (3.26) | 20.52 (6.33) | t = -1.38 | 0.18 |
|  | FWHM | 13.76 (0.80) | 13.68 (0.81) | t = 0.34 | 0.74 |
|  | Fit error | 4.21 (1.01) | 4.08 (1.04) | t = 0.48 | 0.64 |
| MT/V5 | *GABA* |  |  |  |  |
|  | SNR | 16.67 (2.81) | 17.05 (2.97) | t = -0.47 | 0.64 |
|  | FWHM | 17.68 (1.36) | 17.95 (1.20) | w = 250.5 | 0.16 |
|  | Fit error | 4.10 (0.80) | 4.24 (0.86) | w = 303.5 | 0.69 |
|  | *Glx* |  |  |  |  |
|  | SNR | 16.18 (3.17) | 17.61 (3.46) | t = -1.53 | 0.13 |
|  | FWHM | 15.74 (1.33) | 15.35 (1.36) | w = 413.5 | 0.10 |
|  | Fit error | 4.28 (1.06) | 4.10 (0.71) | t = 0.73 | 0.47 |

**Table S2** Results of the post-hoc analyses on the behavioral data comparing performance between training days.

|  | Sample 1 | | Sample 2 | | | t | *df* | *p_corr_* |
| --- | --- | --- | --- | --- | --- | --- | --- | --- |
| **Tests in FBW condition** | |  | |  |  | | | |
| TA-VFB group | Day 1 | | Day 2 | | | 5.81 | 22 | < 0.001*** |
|  | Day 1 | | Day 3 | | | 9.7 | 25 | < 0.001*** |
|  | Day 1 | | Day 4 | | | 9.24 | 25 | < 0.001*** |
|  | Day 1 | | Day 5 | | | 9.35 | 24 | < 0.001*** |
|  | Day 2 | | Day 3 | | | 5.18 | 22 | 0.001*** |
|  | Day 2 | | Day 4 | | | 5.05 | 22 | 0.001*** |
|  | Day 2 | | Day 5 | | | 5 | 22 | 0.001*** |
|  | Day 3 | | Day 4 | | | 0.0576 | 25 | 9.55 |
|  | Day 3 | | Day 5 | | | - 0.372 | 24 | 7.13 |
|  | Day 4 | | Day 5 | | | - 0.484 | 24 | 6.33 |
| CA-VFB group | Day 1 | | Day 2 | | | 3.87 | 24 | 0.007** |
|  | Day 1 | | Day 3 | | | 4.73 | 24 | < 0.001*** |
|  | Day 1 | | Day 4 | | | 7.08 | 23 | < 0.001*** |
|  | Day 1 | | Day 5 | | | 6.9 | 23 | < 0.001*** |
|  | Day 2 | | Day 3 | | | 2.58 | 24 | 0.16 |
|  | Day 2 | | Day 4 | | | 4.35 | 23 | 0.0025** |
|  | Day 2 | | Day 5 | | | 3.63 | 23 | 0.014 |
|  | Day 3 | | Day 4 | | | 2.32 | 23 | 0.297 |
|  | Day 3 | | Day 5 | | | 1.47 | 23 | 1.55 |
|  | Day 4 | | Day 5 | | | - 1.28 | 22 | 2.14 |
| **Training in FB condition** | |  | |  |  | | | |
| TA-VFB group | Day 1 | | Day 2 | | | 7.97 | 22 | < 0.001*** |
|  | Day 1 | | Day 3 | | | 9.24 | 21 | < 0.001*** |
|  | Day 1 | | Day 4 | | | 9.3 | 22 | < 0.001*** |
|  | Day 1 | | Day 5 | | | 11.2 | 21 | < 0.001*** |
|  | Day 2 | | Day 3 | | | 5.06 | 22 | < 0.001*** |
|  | Day 2 | | Day 4 | | | 5.09 | 23 | < 0.001*** |
|  | Day 2 | | Day 5 | | | 7.56 | 21 | < 0.001*** |
|  | Day 3 | | Day 4 | | | 2.07 | 23 | 0.05 |
|  | Day 3 | | Day 5 | | | 6.18 | 22 | < 0.001*** |
|  | Day 4 | | Day 5 | | | 3.51 | 22 | 0.02 * |
| CA-VFB group | Day 1 | | Day 2 | | | 7.9 | 23 | < 0.001*** |
|  | Day 1 | | Day 3 | | | 8.57 | 23 | < 0.001*** |
|  | Day 1 | | Day 4 | | | 9.8 | 23 | < 0.001*** |
|  | Day 1 | | Day 5 | | | 8.68 | 23 | < 0.001*** |
|  | Day 2 | | Day 3 | | | 5.22 | 24 | < 0.001*** |
|  | Day 2 | | Day 4 | | | 8.35 | 24 | < 0.001*** |
|  | Day 2 | | Day 5 | | | 4.07 | 24 | 0.0045** |
|  | Day 3 | | Day 4 | | | 5.74 | 24 | < 0.001*** |
|  | Day 3 | | Day 5 | | | 0.451 | 24 | 6.56 |
|  | Day 4 | | Day 5 | | | - 3.86 | 24 | 0.0075** |

CA-VFB group, concurrent augmented visual feedback group; TA-VFB group, terminal augmented visual feedback group; FB condition, feedback condition; FBW condition, feedback withdrawal condition;

* *p_corr_* ≤ 0.05, ** *p_corr_* < 0.01; *** *p_corr_* < 0.001.

**Table S3** Results of the post-hoc analyses, comparing MRS-assessed levels of GABA+ and Glx between different brain regions.

|  | VOI 1 | VOI 2 | t | *df* | *p*_corr_ |
| --- | --- | --- | --- | --- | --- |
| GABA+ |  |  |  |  |  |
|  | DLPFC | M1 | - 9.04 | 56 | < 0.001*** |
|  | DLPFC | S1 | - 8.61 | 56 | < 0.001*** |
|  | DLPFC | MT/V5 | 6.9 | 56 | < 0.001*** |
|  | M1 | S1 | - 0.118 | 56 | 0.906 |
|  | M1 | MT/V5 | 14.9 | 56 | < 0.001*** |
|  | S1 | MT/V5 | 14.7 | 56 | < 0.001*** |
| Glx |  |  |  |  |  |
|  | DLPFC | M1 | 11.9 | 55 | < 0.001*** |
|  | DLPFC | S1 | 5.85 | 56 | < 0.001*** |
|  | DLPFC | MT/V5 | 0.86 | 56 | 0.389 |
|  | M1 | S1 | - 6.29 | 55 | < 0.001*** |
|  | M1 | MT/V5 | - 9.04 | 55 | < 0.001*** |
|  | S1 | MT/V5 | - 4.73 | 56 | < 0.001*** |

DLPFC, dorsolateral prefrontal cortex; M1, primary motor cortex; MT/V5, medial temporal visual cortex; S1, primary somatosensory cortex.

*** *p_corr_* < 0.001.

**Table S4** Results of the multiple linear regression model predicting later learning gain using baseline levels of GABA+

| Group | R^2^ | R^2^-adj | F | Predictor | B (SE) | t_(20)_ | *p*-value |
| --- | --- | --- | --- | --- | --- | --- | --- |
| Combined group | 0.19 | 0.022 | F_(9,41)_ = 1.13  *p* = 0.366 | intercept | 0.54 (0.32) | 1.71 | 0.09 |
|  |  |  |  | M1-GABA+ | 0.20 (1.43) | 0.14 | 0.89 |
|  |  |  |  | DLPFC-GABA+ | - 0.96 (1.81) | - 0.53 | 0.60 |
|  |  |  |  | S1-GABA+ | 0.18 (2.00) | 0.09 | 0.93 |
|  |  |  |  | MT/V5-GABA+ | 1.58 (1.12) | 1.41 | 0.17 |
|  |  |  |  | Group [CA-VFB] | - 1.07 (0.45) | - 2.38 | 0.02^*^ |
|  |  |  |  | M1-GABA x Group [CA-VFB] | - 2.66 (2.38) | - 1.11 | 0.27 |
|  |  |  |  | DLPFC-GABA x Group [CA-VFB] | 0.56 (2.27) | 0.25 | 0.81 |
|  |  |  |  | S1-GABA x Group [CA-VFB] | 1.13 (2.46) | 0.05 | 0.96 |
|  |  |  |  | MT/V5-GABA x Group [CA-VFB] | - 1.45 (2.07) | - 0.70 | 0.49 |

CA-VFB group, concurrent augmented visual feedback group; DLPFC, dorsolateral prefrontal cortex; M1, primary motor cortex; MT/V5, medial temporal visual cortex; S1, primary somatosensory cortex; B = regression coefficient; SE = standard error; R^2^-adj = adjusted R^2^.

* *p*-value ≤ 0.05

**Table S5** Results of the multiple linear regression model predicting later learning gain using baseline levels of Glx

| Group | R^2^ | R^2^-adj | F | Predictor | B (SE) | t_(20)_ | *p*-value |
| --- | --- | --- | --- | --- | --- | --- | --- |
| Combined group | 0.25 | 0.088 | F_(9,40)_ = 1.52  *p* = 0.173 | intercept | 0.56 (0.33) | 1.69 | 0.09 |
|  |  |  |  | M1-Glx | - 0.12 (0.56) | - 0.21 | 0.84 |
|  |  |  |  | DLPFC-Glx | 0.25 (0.52) | 0.48 | 0.63 |
|  |  |  |  | S1-Glx | 0.07 (0.62) | 0.12 | 0.91 |
|  |  |  |  | MT/V5-Glx | 0.05 (0.43) | 0.12 | 0.90 |
|  |  |  |  | Group [CA-VFB] | - 0.99 (0.48) | - 2.07 | 0.04* |
|  |  |  |  | M1-Glx x Group [CA-VFB] | 1.04 (0.93) | 1.12 | 0.27 |
|  |  |  |  | DLPFC-Glx x Group [CA-VFB] | - 0.57 (0.81) | - 0.708 | 0.48 |
|  |  |  |  | S1-Glx x Group [CA-VFB] | 0.46 (0.81) | 0.57 | 0.57 |
|  |  |  |  | MT/V5-Glx x Group [CA-VFB] | - 1.42 (0.76) | - 1.86 | 0.07 |

CA-VFB group, concurrent augmented visual feedback group; DLPFC, dorsolateral prefrontal cortex; M1, primary motor cortex; MT/V5, medial temporal visual cortex; S1, primary somatosensory cortex; B = regression coefficient; SE = standard error; R^2^-adj = adjusted R^2^.

* *p*-value ≤ 0.05

**Figure S1** Heatmap of the locations of MRS VOIs and the MRS Spectra obtained from these VOIs in the two groups


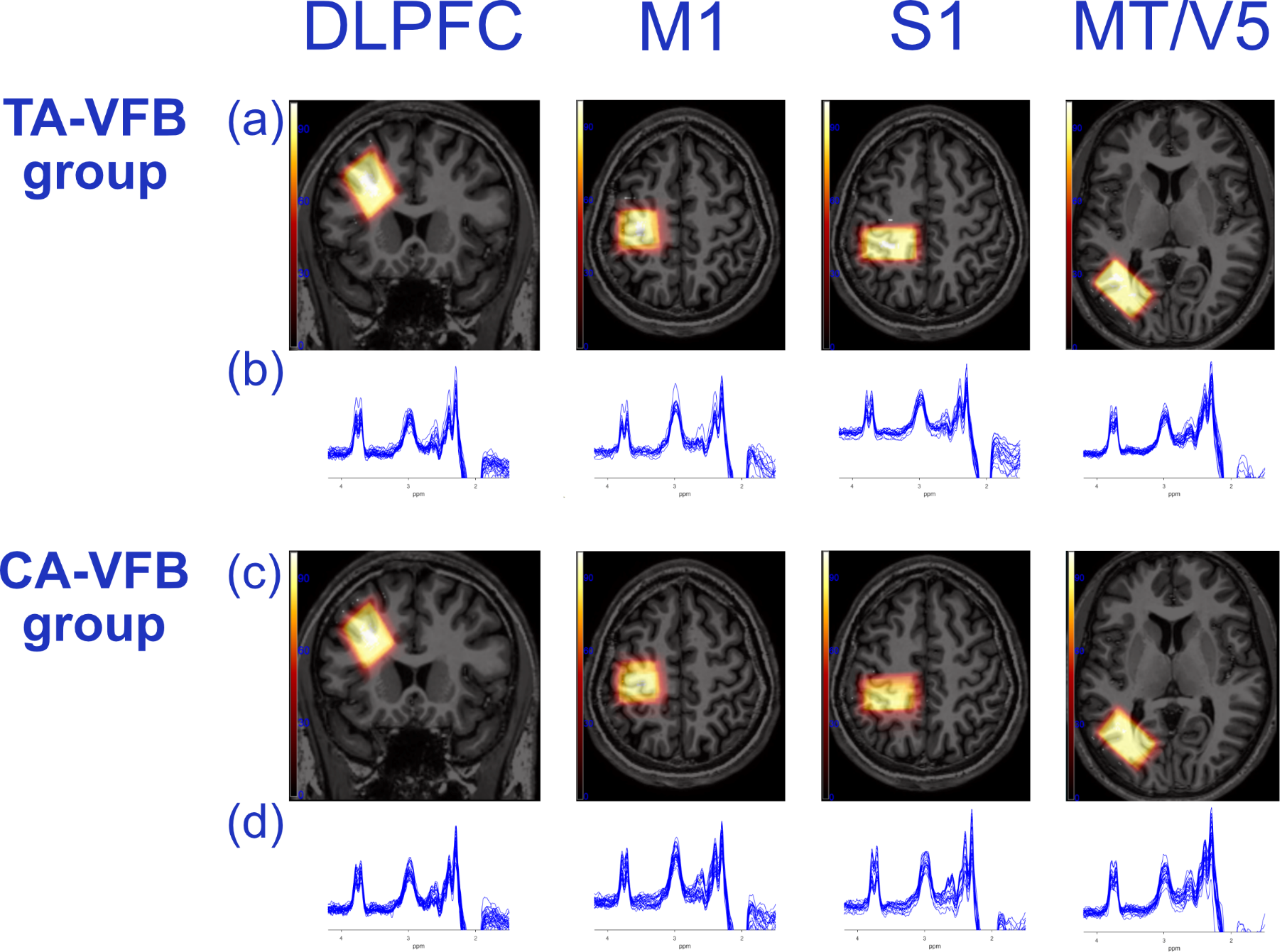


**Figure S1** Heatmap of the locations of the MRS VOIs and the MRS Spectra obtained from each MRS VOI in the two experimental groups. (a) and (c) The heatmap, which is overlaid on an anatomical MRI from the study sample co-registered to the MNI space, shows the MRS VOIs overlap across participants within each group, with brighter (yellow) colors representing greater overlap and darker (red) colors representing less overlap. (b) and (d) Edited spectra from the obtained four brain areas (M1, S1, DLPFC, MT/V5) of the participants in each group.

**Figure S2** Overlap between the M1 and S1 voxels in each separate group.


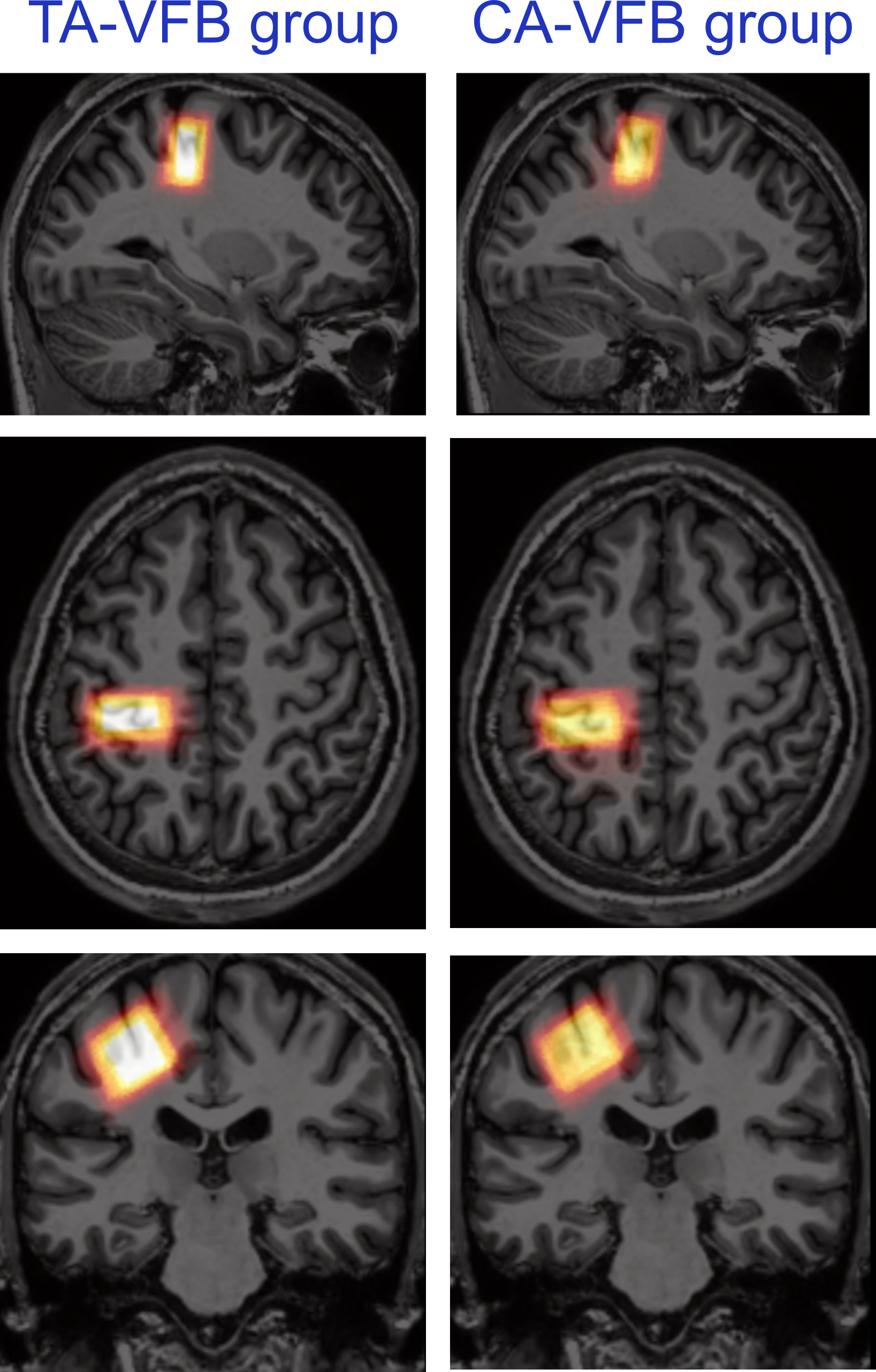


**Figure S2** Overlap between the M1 and S1 voxels in each separate group.
